# Gamma Knife radiosurgery for cerebral arteriovenous malformations: a systematic review and meta-analysis

**DOI:** 10.1007/s10143-022-01751-1

**Published:** 2022-02-18

**Authors:** Musa China, Amisha Vastani, Ciaran Scott Hill, Cornel Tancu, Patrick J. Grover

**Affiliations:** 1 Division of Medicine, University College London (UCL), London, UK; 2 Department of Neurosurgery, National Hospital for Neurology and Neurosurgery, Queen Square, London, UK; 3 Cancer Biology Division, The UCL Cancer Institute, University College London, London, WC1E 6DD UK

**Keywords:** Arteriovenous malformation, AVM, Gamma knife radiosurgery, Stereotactic radiosurgery, Intracranial haemorrhage, Radiation-induced changes

## Introduction

Cerebral arteriovenous malformations (AVMs) are rare vascular lesions that rupture at an approximate annual rate of 2–4% [1]. Annual haemorrhage rates range from < 1%, for unruptured superficially located AVMs, up to 33% in ruptured AVMs with deep, location and venous drainage [12, 68]. Once haemorrhage occurs, the probability of experiencing new neurological deficit has been reported to be up to 50% and fatality ~ 10% [28, 66]. Gamma knife radiosurgery (GKRS) is an established intervention predominantly favoured for small (< 3.5 cm), surgically high-risk or complex AVMs. Successful AVM GKRS is contingent on abolishing the risk of intracranial haemorrhage (ICH) via complete nidal obliteration, whilst limiting the development of new neurological deficit from radiation-induced changes (RICs) [9, 26, 69].

The complications of AVM GKRS are mainly two-fold. Firstly, patients remain at risk of haemorrhage during the latency period between GKRS and nidus obliteration. The relative risk of AVM haemorrhage during this latency period versus the natural history of an untreated AVM remains a topic of debate [26, 28]. Secondly, RICs can occur following radiosurgery, influenced by AVM and treatment parameters including AVM volume, location and treatment dose [12]. Approximately 34% of patients develop RICs, ~ 8% of patients develop RIC-related neurological symptoms and ~ 3% will experience permanent neurologic deterioration. However, considerable variations in the reported rates, durations and definition of RICs still remain. Further, delayed adverse effects including radiation necrosis and cyst formation following GKRS have been reported, yet there is a paucity of literature conclusively describing their incidence [38].

A previous systematic review and meta-analysis quantified haemorrhage risk and obliteration rate following AVM stereotactic radiosurgery (SRS), but it had limitations [72]. The review included 69 observational studies, of which, however, approximately only a third solely treated AVMs with GKRS; the majority reporting on linear accelerator (LINAC) or other SRS-modalities. Additionally, the median duration of study follow-up was less than three years; an arguably limited time-frame to comprehensively analyse clinical outcomes of AVM GKRS, which evolve in a time-dependent manner over a latency period of 2–3 years following radiosurgical intervention [1, 12, 68].

Alongside imaging advancements and increasingly sophisticated radiation delivery systems since the inception of GKRS, there has also been an increasing knowledge base on AVM obliteration, RICs and post-GKRS ICH [12]. Whilst there are several studies that review or report on these outcomes, none are both systematic or comprehensive. We sought to systematically review the current literature and comprehensively quantify the efficacy: complication profile of GKRS for cerebral AVMs.

## Materials and methods

### Registration and reporting standards

We performed this systematic review following the Preferred Reporting Items for Systematic Reviews and Meta-Analyses (PRISMA) guidelines (Appendix 1) [53]. The study protocol is published on PROSPERO (CRD 42,021,285,118).

### Search strategy

We performed a comprehensive literature search (Appendix 2) using OVID Medline, EMBASE, ClinicalTrials.gov, OpenGrey and Cochrane Library from 1st January 1989 to 1st September 2021 for relevant articles. We reviewed the bibliographies of included studies for further articles meeting our eligibility criteria.

### Eligibility criteria

We sought randomised trials and observational cohort studies, published in English in peer-reviewed journals reporting 20 + adult (18 +) patients with AVMs, diagnosed by MRI or histopathological examination, treated with single-session GKRS. We included studies describing all the following patient and AVM characteristics: (1) median (or mean) margin dose, (2) AVM volume (or maximum nidus diameter), (3) clinical presentation, (4) AVM Spetzler-Martin grades. We included studies reporting all the following clinical and radiological outcomes, with a minimum follow-up period of 12 months following GKRS: (1) complete nidus obliteration rate (angiography or angiography/MRI-confirmed), (2) post-GKRS ICH, (3) RICs or adverse radiation effects (ARE).

### Study selection

Two investigators (MC and AV) independently screened all titles and abstracts for eligibility. The full text of eligible studies was reviewed for inclusion. CSH, CT and PG acted as mediators in cases of disagreement. If multiple studies with overlapping data from a single institution were published, the study reporting the largest sample size and/or the most recent study were prioritised (Appendix 3 and 4).

### Data extraction

Data extraction was performed independently by two authors (MC and AV) from each unique study cohort undergoing GKRS to ensure consistent extraction of patient, AVM and treatment characteristics. We extracted the following outcomes: complete nidus obliteration rate, ICH, radiologic evidence of RIC, symptomatic RIC events (transient or permanent), radionecrosis, cyst formation, radiation-induced neoplasm, seizures (new or worsening), deaths attributed to AVM/GKRS (case-related) and all-cause death rate.

We used outcomes as described per patient. The proportion successfully obliterated was calculated as a proportion of all patients with available radiological follow-up and was defined as the presence of angiographically demonstrated complete obliteration and/or angiography-or-MRI confirmed obliteration. Post-GKRS ICH was defined as any AVM-related haemorrhage detected through appropriate follow-up radiological imaging. RICs were classified as follows: (1) total radiologic RIC: any MRI evidence of peri-nidal T2-weighted hyperintensities after GKRS; (2) transient symptomatic RIC: radiologic RIC which correlated with new or worsening neurological symptoms, typically headache, seizure or focal neurological deficit, which resolved by the end of study follow-up; and (3) permanent RIC: symptomatic RIC without full recovery to pre-GKRS neurological baseline at the end of study follow-up.

### Risk of bias

Two authors (MC and AV) assessed the risk of bias of each cohort as serious, moderate, low, or unclear risk according to the following domains of the Cochrane ROBINS-I tool [70]: (1) confounding bias, (2) selection bias, (3) bias in classification of interventions, (4) bias due to deviations from intended interventions, (5) attrition bias, (6) detection bias and (7) selective outcome reporting (Appendix 5).

### Statistical analysis

We quantified the distribution of cohort-level characteristics with descriptive analyses. We calculated the incidence of clinical outcomes as a proportion of total patients treated with GKRS. We quantified the occurrence of haemorrhage and fatality at any time after GKRS either during the total person-years of follow-up stated or by multiplying the median (or mean) follow-up duration by total number of patients treated. We calculated annual incidence rates for haemorrhage and fatality, using Poisson distributions.

We aimed to perform the following subgroup analyses: (1) clinical and obliteration outcomes reported in studies stratifying by AVM Spetzler-Martin grades [67]: I–II, III and IV–V; (2) clinical and obliteration outcomes reported in studies stratifying by Radiosurgery-Based AVM Score [61, 62].

A meta-analysis was performed for all primary outcomes. DerSimonian-Laird random effects models were used for all summary effect estimates with a Freeman-Tukey double arcsine transformation [11, 25].

Heterogeneity was assessed using the quantity *I*^2^ [34]. Heterogeneity was investigated using Baujat plots [3] and leave-one-out sensitivity analysis. Publication bias was assessed using Funnel plots and Egger’s test was performed to assess Funnel plot symmetry.

Moderator analysis was performed using meta-regression techniques to determine association of patient, AVM and treatment characteristics with clinical and radiological outcomes. Patients were stratified by the following pre-specified variables: age, sex, study mid-year, median margin treatment dose (Gy), median AVM volume (cm^3^), deep location (%), eloquent location (%), deep venous drainage (%).

R version 4.0.4 (The R Foundation for Statistical Computing, Vienna, Austria) was used for all statistical analyses. For all statistical tests, *P* < 0.05 was considered statistically significant.

## Results

### Study selection

**Fig. 1:**
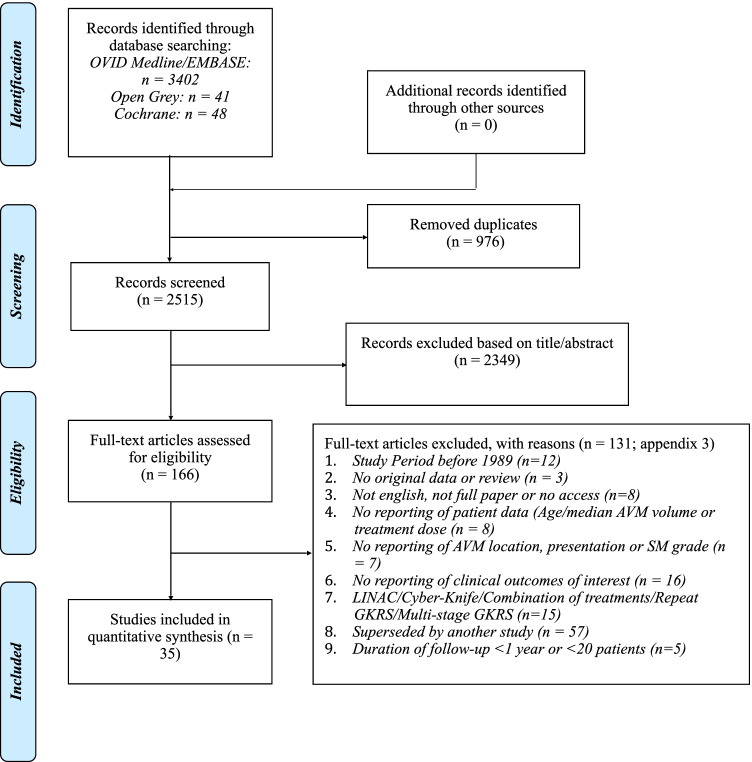
PRISMA flowchart of search strategy used for the systematic review. Arteriovenous malformation (AVM), gamma-knife radiosurgery (GKRS), linear accelerator (LINAC), Spetzler-Martin (SM)

After screening 3402 publications, 166 full-text studies were reviewed with 34 studies included in final analysis (Fig. 1, Appendix 3, 4). A detailed summary of included studies is presented in Appendix 6.

### Study characteristics

In the 35 cohorts (34 studies) receiving GKRS, including a total of 8673 patients with 41,544 patient-years of follow-up, the median cohort-level patient characteristics were: population size 182 patients, age at initial treatment 35 years, 53% male, follow-up duration 60 months, 49% presented with ICH, 71% AVMs were lobar, 20% deep and 72% in eloquent locations (Table 1 and Appendix 6).

**Table 1:** Summary characteristics of all studies: single-stage gamma knife radiosurgery (GKRS) intervention for brain arteriovenous malformations (AVMs)

Characteristics	Median (Interquartile range)	Cohorts reporting characteristics	Patients reported
Demographics			
Number of patients	182 (98–278)	35	8673
Age, years	35.1 (30.9–40.0)	35	8673
Male:Female, %	53:47	35	8673
Duration of Follow-up, mo	60.2 (37.0–78.8)	33^a^	8355
Follow-up person-years, y	547.3 (244.1–1555)	33^a^	8355
Mid-year study period	2001 (1998–2005)	35	8673
Presentation, %			
Ruptured AVM	49.3 (36–58.8)	35	8673
Clinical presentation: seizures^b^	23 (18.8–31.5)	27	4931
Clinical presentation: incidental finding	8 (3–14)	19	3697
Previous intervention, %			
Embolisation	13.4 (2.3–22.6)	33	8436
Surgical resection	6.7 (2.5–9.7)	28	7842
Previous Radiosurgery (including GKRS/LINAC/CK)	0 (0–1.2)	34	8436
Angioarchitecture			
Nidus Volume^c^, cm^3^	3.9 (2.6–4.9)	33	8595
Nidus Diameter Max., cm	2.3 (2.0–2.8)	20	6430
Venous Drainage^a^			
Superficial only	44 (34.6–67.2)	12	4835
Deep	56 (32.9–65.4)	13	5170
Associated Aneurysm^f^, %	10.4 (6.7–14.6)	15	4320
Location, %			
Eloquent, %	71.6 (59.8–91.5)	16	5407
Deep^a^, %	19.5 (12.7–35.7)	31	8116
Basal Ganglia/Thalamus	8.5 (4–15.2)	19	3312
Brainstem	3.5 (0.7–11.8)	19	3312
Lobar^g^, %	71.3 (52.7–82.9)	27	7730
Cerebellum, %	7.3 (0–8.8)	21	3919
Spetzler–Martin grade			
SM1, %	11.8 (4.5–17.2)	30^*d*^	7586
SM2, %	32.9 (21–39.3)	30^*d*^	7586
SM3, %	36.6 (29.4–45.8)	32^*e*^	7640
SM4, %	9.8 (1.7–14.7)	29^*e*^	6994
SM5, %	0 (0–1.9)	29^*e*^	6994
SM6, %	0 (0–0)	29^*e*^	6994
RBAS	1.4 (1.2–1.5)	16	4780
Treatment characteristics			
Marginal GK dose, Gy	20 (19–22)	35	8673
Maximum GK dose, Gy	37.9 (36–40)	19	3679
Repeat GK performed, %	13.2 (6.8–18.7)	26	5461

Arteriovenous malformation (AVM), cyber-knife (CK), gamma-knife/radiosurgery (GK/RS), linear accelerator (LINAC), radiosurgery-based AVM Score (RBAS), Spetzler-Martin (SM)

^a^Follow-up missing: Franzin et al. 2013 [24] and Nicolato et al. 2002 [54] did not include median FU duration, except including minimum FU duration (see Appendix 6). Pollock et al. 2016 [64] grouped both cohorts 1990–1999 and 1999–2009 together for a total 2966 patient years

^b^Seizure presentation missing: Orio et al. 2006 [56], Hirschmann et al. 2019 [35], Hasegawa et al. 2018 [32], Bir et al. 2015 [4], Chen et al. 2018 [7], Kano et al. 2012 [41], Pollock et al. 2016 [64] (1990–1999 cohort)*,* Pollock et al. 2016 [64] (1999–2009 cohort)

^c^Volume missing: Ditty et al. 2017[18]

^d^SM-grade not included and not possible to calculate according to data provided within these select papers. Pollock et al. 2016 [64] 1990–1999/1999–2009 cohorts and Hirschmann et al. 2019 [35] reported Spetzler-Ponce classification which did not stratify SM 1/2 or SM 4/5 grade AVMs further. Orio et al. 2006 [56] and Zhao et al.2008 [75] did not report venous drainage

^e^In addition to the previously stated papers, Hasegawa et al. 2018 [32] does not stratify individually but groups SM 3-5 AVMs frequency (55%)

^f^Includes both flow-related and intra-nidal aneurysm

^*g*^Includes all AVMs labelled as Hemispheric/lobar/superficial without any further analysis

Thirty-three (97%) studies were from single centres and one (3%) was multi-centre (Appendix 7). Thirteen (39%) studies were from North America [4, 14–16, 18, 41, 43–45, 52, 56, 66, 74], 12 (36%) from Asia [5, 6, 8, 31, 32, 36, 39, 46, 47, 51, 57, 75] and 8 (24%) from Europe [2, 24, 35, 49, 54, 58, 65, 71].

Overall, included studies were at moderate risk of bias (Appendix 5). All studies were at moderate risk of bias due to confounding as none of the studies were randomised and none concealed treatment allocation. Fifteen studies (44%) were at moderate risk of selection bias; most reasoned to the retrospective selection of patients with a minimum of 2 years of follow-up. No studies were at risk of bias due to classification of intervention or from performance bias from deviation of intended intervention. Four studies (12%) were at moderate risk of attrition bias for obliteration outcomes from loss of patients to follow-up [6, 8, 54, 57]. All four studies had > 80% of total patients treated available for analysis at the end of follow-up. All studies were retrospective, except one which identified and followed consecutive patients prospectively (Appendix 7) [65]. Three studies (9%) were deemed moderate risk of reporting bias [6, 8, 56]. Overall, no studies were deemed to be at serious risk of bias for any of the seven domains of the ROBINS-I tool [70].

### Outcomes after GK

Of 35 cohorts with a total of 8673 patients (Table 2), 576 (6.6%) patients experienced an intracranial haemorrhage event, at a median time interval of 20 months following AVM GKRS. Total RIC events occurred in a median 29.8% (range, 0–63.3%) of patients. Transient symptomatic RICs occurred in a median of 6.3% (range, 0–17.1%) of patients. Permanent symptomatic RICs occurred in a median of 2.7% (range, 0–14.4%) of patients. Case-related deaths (secondary to ICH or RIC) occurred in 88 (2.1%) patients. New-onset or increased frequency of seizures occurred in a median of 1.8% (range, 0–11.8%) of patients.

**Table 2:** Outcomes/incidence rate following single-stage gamma knife radiosurgery for brain arteriovenous malformations

Outcomes	Cohorts, *n* (/35)	Patients	Number of outcome events/total no. of patients (%)	Median rate, % (range)	Number of outcome events/total person-years^a^	Estimate annual incidence, % (95% CI) per 100 person-years
Haemorrhage	35^a^	8673	576/8673 (6.64%)	6.2% (2.01–18.18)	576/41554	1.38 (1.28–1.50)
				Median months post-GKRS: 19.7		
Total RIC	18	4369	1268/4369 (29.0%)	29.8% (0–63.3)		
Transient symptomatic RIC	22^f^	5685	339/5685 (5.96%)	6.29% (0–17.07)		
Permanent symptomatic RIC	28^ g^	6961	175/6961 (2.51%)	2.67% (0–10.45)		
Mortality (2° to ICH/RIC)	23^c^	4240	88/4240 (2.08%)	1.89% (0.38–7.46)	88/19075	0.46 (0.37–0.57)
Mortality (all-cause)	24	6401	212/6401 (3.31%)	2.36% (0.42–14.93)	212/31483	0.67 (0.58–0.76)
Seizure (new-onset or increased frequency)	17	3385	104/3385 (3.07%)	1.76% (0–11.8)		
Radionecrosis	8	1010	22/1010 (2.18%)	2.5% (0–6.9)		
Cyst Formation/encapsulated haematoma^e^	15	3446	70/3446 (2.03%)	1.18% (0–5.91)		
Radiation-induced neoplasm	4	946	1/946 (0.11%)	0% (0–0.34%)		

Gamma-knife radiosurgery (GKRS), intracranial haemorrhage (ICH), radiation-induced changes (RIC)

^a^Total person years at risk of haemorrhage: sum of person-years of follow-up described or by multiplying the median (or mean if median not provided) follow-up duration by total number of treated patients. Franzin et al. 2013 [24] and Nicolato et al. 2002 [54] follow-up stated as ‘*minimum 36 months*’ and ‘*6 months minimum*’. Total haemorrhage risk patient-years follow up for all 32 cohorts was calculated with the median follow up person-years average of 32 cohorts (529.15) assumed for Franzin et al. 2013 and Nicolato et al.2002. Pollock et al. 2016 [64] grouped both cohorts 1990–1999/1999–2009 together for haemorrhage and RICs which has been accounted for

^b^Hirschmann et al. 2019 [35], grouped ‘*radiologically diagnosed oedema or late onset cyst formation with or without new neurological symptoms*’ under one total RICs group, with no separation into transient or permanent. Hu et al. 2020 [36], grouped all RICs in one total RIC group, with no separation of transient or permanent. Pollock et al. 2016 [64] grouped both cohorts 1990–1999 and 1999–2009 together for Haemorrhage and RICs which has been accounted for in the table

^c^Chen et al. 2018 [7] does not specify cause of deaths

^d^Missing Transient RIC data: Parkhutik et al. 2013 [56], Pollock et al. 2016 [64] 1990–1999, 1999–2009 cohort, Franzin et al. 2013 [24], Orio et al. 2006[56]

^e^Cyst Formation includes either asymptomatic (radiological-only) or symptomatic presentation

^f^Ding et al. [14–16], Kano et al. [45] classified headache as TRIC event. Ding et al. [14–16] and Kano et al. [45] classified seizure as TRIC event. ^g^Pollock et al. 2016 [64] classified seizure events as permanent RIC events

Annual post-GKRS haemorrhage rate was 1.38% (95% CI 1.28–1.50). Annual case-fatality rate was 0.46% (95% CI 0.37–0.57). Annual all-cause fatality rate (AVM/non-AVM related) was 0.67% (95% CI 0.58–0.76). All annual incidence of outcomes were calculated per 100 person-years of follow-up over the duration of follow-up described in included studies.

Complete nidus obliteration, confirmed on angiography imaging, was achieved in 56.7% (3092/5450) of patients in 21 cohorts, at the end of follow-up after single-stage Gamma knife treatment [5–8, 14–16, 24, 31, 32, 36, 39, 46, 47, 49, 51, 52, 54, 57, 58, 65]. Overall nidus obliteration, confirmed with either angiography or MRI imaging, was achieved in 67.8% (4605/6792) of patients in 29 cohorts, at the end of follow-up after single-stage Gamma knife treatment [2, 4–8, 14–16, 18, 24, 31, 32, 36, 39, 46, 47, 51, 52, 54, 56–58, 64, 65, 71, 74, 75]. Table 3 outlines AVM GKRS obliteration outcomes. Median time to complete nidus obliteration was 35.4 months following GKRS (95% CI 32.5–38.3). Six studies stratified their obliteration outcomes for AVMs of Spetzler-Martin grades I–II, totalling 916 AVMs (study median margin dose 22.5 Gy and median duration follow-up 45.5 months) [8, 16, 45, 52, 65, 71]. Complete nidus obliteration rate was 74.0% (657/888) (Table 4). Six studies stratified their obliteration outcomes for AVMs of Spetzler-Martin grade III, totalling 994 AVMs (study median margin dose 20.6 Gy and median duration follow-up 45.6 months) [8, 15, 41, 52, 65, 71]. Complete nidus obliteration rate was 69.1% (349/505) (Table 5). Six studies stratified their obliteration outcomes for AVMs of Spetzler-Martin grade IV–V, totalling 176 AVMs (study median margin dose 21.4 Gy and median duration follow-up 41.3 months) [2, 8, 14, 47, 52, 65]. Complete nidus obliteration rate was 32.4% (57/176) (Table 6). We were unable to consistently and reliably quantify clinical outcomes according to Spetzler-Martin grade because most studies did not stratify their reporting of outcomes by SM grade. We were unable to consistently and reliably quantify outcomes according to RBAS score because studies did not stratify their reporting of outcomes by the RBAS score, of which the median was 1.42 (IQR 1.2–1.5).

**Table 3:** Obliteration rates following single-session GKRS for brain AVMs

Obliteration rate	Angiography confirmed	Cohorts, *n*	Angiography or MRI-confirmed	Cohorts, *n*
Obliteration Rate (*n*/total patients)	56.7% (3092/5450)	21	67.80% (4605/6792)	29
Meta-analysis pooled estimate	60.5% (54.2–66.7)	21	69.68% (65.89–73.48)	29
Median obliteration rate	58.3% (33.6–87.8)	21	69.80% (42.42–87.80)	29
Median obliteration rate (cohorts with minimum 2 years follow-up)	63.5% (33.6–87.8)	14	70.85% (42.42–87.79)	19

Arteriovenous malformation (AVM), gamma-knife radiosurgery (GKRS), magnetic resonance imaging (MRI)

**Table 4:** Overview of AVM obliteration rates reported in studies stratified by Spetzler-Martin grade I and II

Spetzler-Martin grade I/II (6 studies)										
Study	Study total patient no	Number of SM I-II AVMs	SM grades (%)	Study Median age^c^ (range/SD)	Study median nidus volume^c^ cm^3^ (Range)	Study median prescription dose^c^ Gy (range)	Study median duration follow-up^c^, mo (range)	SM I/II complete obliteration rate—angiography-confirmed (%)	SM I/II complete obliteration rate—(angiography or MRI)	Study median time to obliteration, mo (range)
Ding et al. 2014 [15]	502	502	I 147 (29.3%) II 355 (70.7%)	35.2 (4.1–81.8)	2.4 (0.1–22.5)	23 (7–36)	61.6 (6.8–239.4)	304/502 (60.6%)	382/502 (76.1%)	39.5 (5.7–192.8)
Kano et al. 2012 [43]	217	217	I 34 (16%) II 183 (84%)	38 (3–77	2.3 (0.1–14.1)	22 (15–27)	64 (6–267)	Actuarial obliteration rates: 3/4/5/10 years were 41%, 66%, 77%, and 83%	148/217 (68.2%)	*37 (95% CI 36–39)
Zeiler et al. 2011 [72]	41	16	I 7 (43.7%) II 9 (56.3%)	40.9 (14–74)	5.05	20.3 Gy (16 – 26.4)	43.1		15/16 (93.75%)	27.6
Choe et al. 2008 [8]	100	45	I 18 (40%) II 27 (60%)	34 (5–66)	4.3(0.1–29.3)	20.8 (13–32)	37.5 (5–63)	^a^12/17 (70.59%)	^a^12/17 (70.59%)	25.3 (6–43)
Tuleasca et al. 2021 [69]	149	113	I 42 (37.2%) II 71 (62.8%)	40 (18–68)	2 (0.09–10)	24 (18–25)	48 (12–154)		80/113 (71%)	36 (12–96)
Raboud et al. 2018 [63]	64	23	I 9 (39.1%) II 14 (60.9%)	46 (13–79)	1.2 (0.03–11.3)	24 (18–24)	38 (12–75)		20/23 (87%)	35 (8–56)
Total:		916	I 257 (28.1%) II 659 (71.9%)					^b^316/519 (60.9%)	657/888 (74.0%)	35.5 months (25.3–39.5)

Arteriovenous malformation (AVM), magnetic resonance imaging (MRI), Spetzler-Martin (SM)

^a^Choe et al. 2008 [8] 28 patients lost to follow-up resulting in obliteration rate calculated as proportion of 17 patients with known outcome

^b^Kano et al. 2012 [45] was excluded from summative quantitative analysis as presents angiography-confirmed obliteration rate as Kaplan–Meier probability rates and with no pre-specified time-point at which to select obliteration rate for overall analysis

^c^Statistics generated based on whole-study parameters (e.g., not necessarily just grade I–II patients)

**Table 5:** Overview of AVM obliteration rates reported in studies stratified by Spetzler-Martin grade III

Spetzler-Martin grade III (6 studies)										
Study	Study total patient no	Number of SM III AVMs	SM grade III (%)	Study median age^e^ (range/SD)	Study median nidus volume^e^ cm3 (range)	Study median prescription dose^e^ Gy (range)	Study Median Duration of follow-up^e^, mo (range)	SM III complete obliteration rate—angiography-confirmed (%)	SM III complete obliteration rate—(angiography or MRI)	Study median time to obliteration, mo (range)
Ding et al. 2014 [14]	398	398	III (100%)	30.9 (3.7–81.1)	2.8 (0.1–27.8)	20 (5–32)	54.3 (5.3–230.4)	222/398 (55.8%)	276/398 (69.4%)	45.5 months
Kano et al. 2014 [39]	474	474	III (100%)	33 (± SD 1.32)	3.8 (0.1–26.3)	20 Gy (13–25)	89 (2–278)	Actuarial obliteration rates: 3/4/5/10 years were 39%, 57%, 59%, 62%	Actuarial obliteration rates: 3/4/5/10 years were 48%, 69%, 72% and 77%	–
Zeiler et al. 2011 [72]	41	21	III (100%)	40.9 (14–74)	5.05	20.3 (16–26.4)	43.1		19/21 (90.5%)	27.6
Choe et al. 2008 [8]	100	36	III (100%)	34 (5–66)	4.3(0.1–29.3)	20.8 (13–32)	37.5 (5–63)	^c^13/21 (61.9%)	^c^13/21 (61.9%)	25.3 (6–43)
Tuleasca et al. 2021 [69]	149	36	III (100%)	40 (18–68)	2 (0.09–10)	24 (18–25)	48 (12–154)		24/36 (66.7%)	36 (12–96)
Raboud et al. 2018 [63]	64	29	III (100%)	46 (13–79)	1.2 (0.03–11.3)	24 (18–24)	39.6 (12–75)		17/29 (58.6%)	35 (8–56)
Total:		994	III (100%)					^d^235/419 (56.1%)	349/505 (69.1%)	35 months (25.3–45.5)

Arteriovenous malformation (AVM), magnetic resonance imaging (MRI), Spetzler-Martin (SM)

^c^Choe et al. 2008 [8] 15 patients lost to follow-up resulting in obliteration rate calculated as proportion of 21 patients with known outcome

^d^Kano et al. 2014 [41] was excluded from summative quantitative analysis as presents angiography-confirmed obliteration rate as Kaplan–Meier probability rates and with no pre-specified time-point at which to select obliteration rate for analysis. ^e^Statistics generated based on whole-study parameters (e.g., not necessarily just grade III patients)

**Table 6:** Overview of AVM obliteration rates reported in studies stratified by Spetzler-Martin grade IV–V

Spetzler-Martin grade IV/V (6 studies)										
Study	Study total patient no	Number of SM 4/5 AVMs	SM grade 4/5 (%)	Study median age^a^ (range/SD)	Study Median Nidus Volume^a^ cm^3^ (range)	Study Median Prescription dose Gy (range)	Study Median Duration of FU^a^, mo (range)	SM IV-V Complete obliteration rate—angiography-confirmed (%)	SM IV-V complete obliteration rate—(angiography or MRI)	Study median time to obliteration, mo (range)
Ding et al. 2014 [14]	110	110	IV 109 (99.1%) V 1 (0.9%)	27.6 (4.7–75.1)	5.7 (1.2–33.0)	19 (10–25)	87.8 (17.3–261.6)	37/110 (33.6%)	48/110 (43.6%)	42.7 (6.2– 223.5)
Choe et al. 2008 [8]	100	19	IV 11 (57.9%) V 8 (42.1%)	34 (5–66)	4.3(0.1–29.3)	20.8 (13–32)	37.5 (5–63)	3/19 (15.8%)	3/19 (15.8%)	25.3 (6- 43)
Arslan et al. 2017 [2]	199	19	IV 14 (73.7) V 5 (26.3)	32 (3–74)	2.5 (0.05–39)	22 (10–26)	60.2 (7–100.1)		1/19 (4%)	–
Kiran et al. 2009 [47]	53	13	IV 11 (84.6) V 2 (15.4)	Mean: 22.7 years (3–55)	Mean: 4.3 (0.1–36.6)	Mean: 23.3 (16–25)	Mean 28 (12–96)	2/13 (15.4%)	2/13 (15.4%)	–
Raboud et al. 2018 [65]	64	11	IV 7 (63.6%) V 4 (36.3%)	46 (13–79)	1.2 (0.03–11.3)	24 (18–24)	39.5 (12–75)		1/11 (9.1%)	37 (8–56)
Zeiler et al. 2011 [74]	41	4	IV 4 (100%) V 0 (0%)	40.9 (14–74)	5.05	20.3 (16–26.4)	43.1		2/4 (50%)	27.6
Total:		176	IV 145 (89.0%) V 18 (11.0%)					42/142 (29.6%)	57/176 (32.4%)	32.3 months (25.3–42.7)

Arteriovenous malformation (AVM); follow-up (FU); gamma-knife (GK); radiosurgery-based AVM score (RBAS); Spetzler-Martin (SM)

^a^Statistics generated based on whole-study parameters (e.g., not necessarily just grade IV–V patients)

### Synthesis of results

**Fig. 2:**
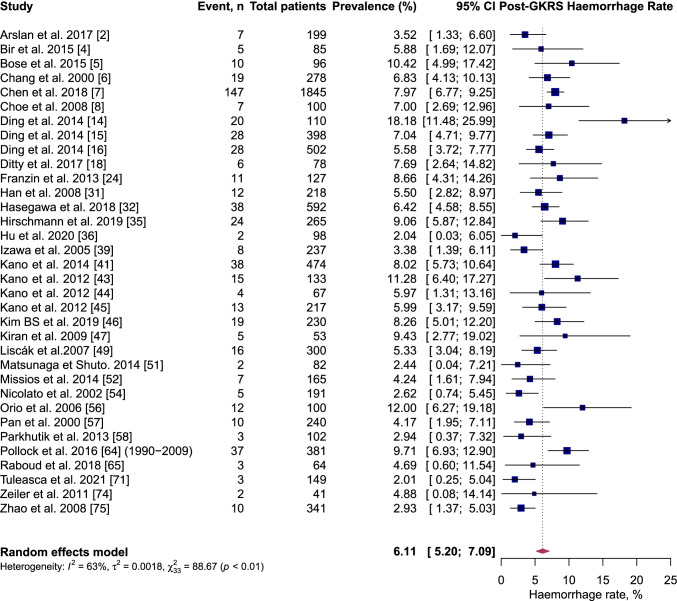
Forest plot: pooled estimates of post-GKRS haemorrhage rate. Arteriovenous malformation (AVM), gamma-knife radiosurgery (GKRS)

**Fig. 3:**
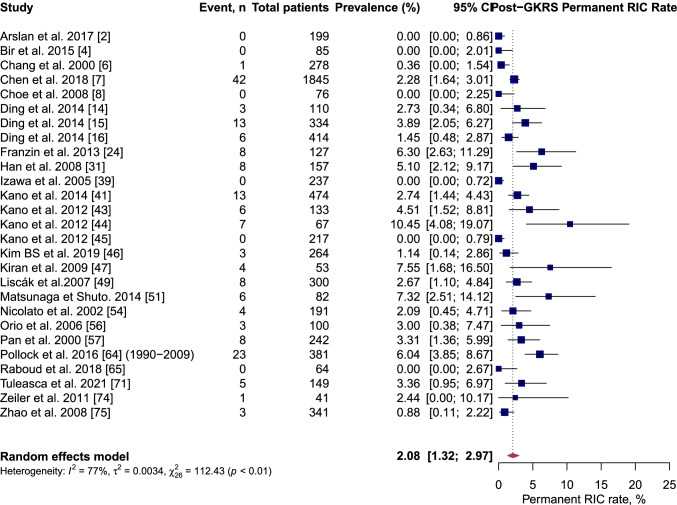
Forest plot: pooled estimates of post-GKRS permanent RICs rate. Arteriovenous malformation (AVM), gamma-knife radiosurgery (GKRS), radiation-induced changes (RICs)

**Fig. 4:**
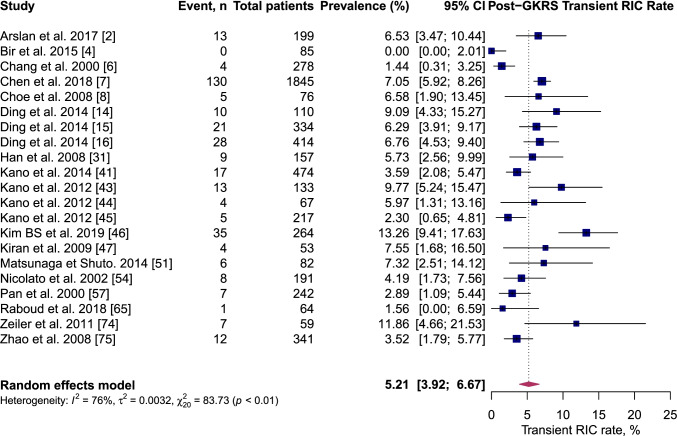
Forest plot: pooled estimates of post-GKRS transient RICs rate. Arteriovenous malformation (AVM), gamma-knife radiosurgery (GKRS), radiation-induced changes (RICs)

On pooled analysis, post-GKRS ICH rate was 6.1% (95% CI 5.2–7.1%, *I*^2^ = 63%) (Fig. 2); permanent symptomatic RIC rate was 2.1% (95% CI 1.3–2.9%, *I*^2^ = 77%) (Fig. 3); transient symptomatic RIC rate was 5.2% (95% CI 3.7–6.7%, *I*^2^ = 76%) (Fig. 4); and case-fatality rate was 2.3% (95% CI 1.7–3.2%, *I*^2^ = 54%).

**Fig. 5:**
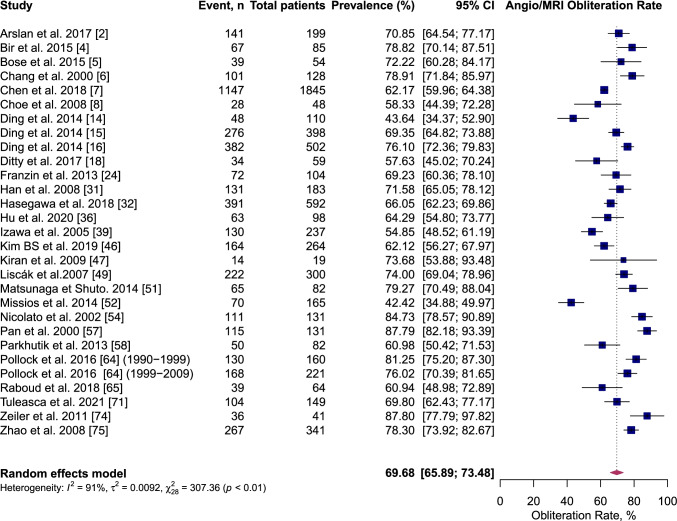
Forest plot: pooled estimates of post-GKRS angiography-or-MRI confirmed obliteration rate. Arteriovenous malformation (AVM), gamma-knife radiosurgery (GKRS), magnetic resonance imaging (MRI)

**Fig. 6:**
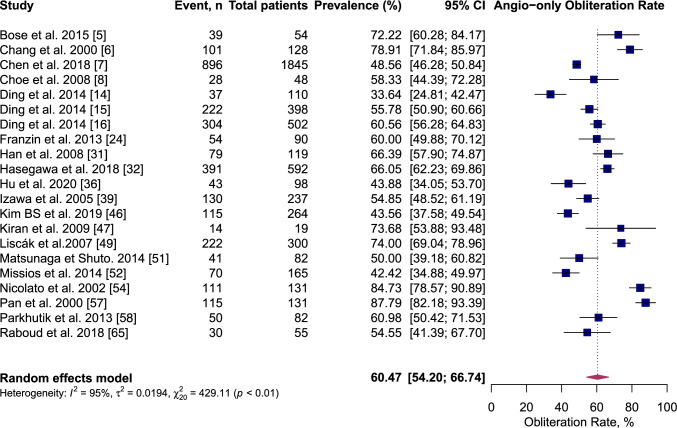
Forest plot: pooled estimates of post-GKRS Angiography-only confirmed obliteration rate. Arteriovenous malformation (AVM), gamma-knife radiosurgery (GKRS), magnetic resonance imaging (MRI)

At the end of follow-up after single-session GKRS, 60.5% (95% CI 54.2–66.7%, *I*^2^ = 95%) of AVMs were confirmed obliterated on angiography imaging and 69.7% (95% CI 65.9–73.5%, *I*^2^ = 91%) of AVMs were confirmed obliterated on either angiography-or-MRI imaging (Figs. 5 and 6). Table 7 summarise the results of meta-analysis.

**Table 7:** Meta-analysis pooled estimates of outcomes post-GKRS for AVMs

Outcome parameter	Summary estimate (95% CI)	*I*^2^
Haemorrhage	6.11% (5.20–7.09)	**63%**
Transient Symptomatic RICs	5.21% (3.92–6.67)	**76%**
Permanent Symptomatic RICs	2.08% (1.32–2.97)	**77%**
Obliteration (Angiography confirmed)	60.47% (54.20–66.74)	**95%**
Obliteration (Angiography or MRI-confirmed)	69.68% (65.89–73.48)	**91%**
Mortality (2° to ICH/RICs)	2.32% (1.66–3.24)	**53.7%**

Arteriovenous malformation (AVM), gamma-knife radiosurgery (GKRS), intracranial haemorrhage (ICH), magnetic resonance imaging (MRI), radiation-induced changes (RIC)

Sensitivity analysis identified one outlier within the haemorrhage pooled analysis [14]. Excluding this outlier yielded a new pooled estimate value of 6.3% (95% CI 5.53–7.27%, *I*^2^ = 51%). One potential outlier was identified within the transient symptomatic RIC pooled analysis [46]. Excluding the outlier, yielded a new pooled estimate value of 4.98% (95% CI 3.8–6.3%, *I*^2^ = 66%).

Cohorts with higher proportions of AVMs with strictly deep venous drainage (*p* = 0.005) and eloquent location (*p* = 0.026) were associated with higher haemorrhage rates. The chance of complete AVM obliteration was higher in more recent cohorts (*p* = 0.021) with younger patients (*p* = 0.018) or AVMs with deep venous drainage (*p* = 0.035). A higher risk of both transient and permanent symptomatic RIC was associated with cohorts with higher proportions of AVMs in deep locations (*p* = 0.002 and 0.005). Appendix 8 summarises moderator analysis performed for primary outcomes.

### Publication bias

Five funnel plots (Appendix 9) illustrate no significant evidence of publication bias. Egger’s regression test for funnel plot asymmetry values: 0.77, 0.89, 0.27, 0.19 and 0.10 for haemorrhage, transient symptomatic RIC, permanent symptomatic RIC, angiography-confirmed and overall obliteration, respectively.

## Discussion

We have systematically reviewed the three primary outcomes defining successful AVM GKRS: rate of AVM obliteration, chance of post-GKRS ICH and risk of radiation-induced complications, to establish a comprehensive and contemporary risk: benefit profile for AVM GKRS.

Starke et al. previously reported on 2236 AVMs treated with GKRS between 1988 and 2013, with mean volume 4.3 cm^3^, 20.5 Gy margin dose and follow-up of 7 years. This multi-centre study reported a comparable overall nidus obliteration rate of 64.7%, haemorrhage rate of 7.4% with annual incidence of 1.1%, whilst symptomatic RICs were transient in 6.7% and permanent in 2.7% of patients at the end of follow-up [69]. The most contemporary systematic review of SRS for brain AVMs was performed by van Beijnum et al. in 2013, investigating the outcomes following treatment of 9436 AVMs across multiple interventions (surgery, SRS or embolisation). They reported a median post-SRS haemorrhage rate of 5.8% and an annual ICH and case-related fatality rate of 1.7 and 0.5 per 100 person-years [72].

Generally, nidus obliteration can be achieved in 65–85% of patients after a 3–5 year latency period [20, 23, 60, 61, 69]. Of note, van Beijnum et al. reported a significantly lower median angiography-confirmed obliteration rate of 38% following AVM SRS [72]. This can partly be reasoned to their strict calculation of obliteration rate, calculated as a proportion of patients confirmed obliterated as a proportion of all SRS-treated patients, which did not account for patients without complete angiography radiological follow-up to confirm obliteration or those patients lost to follow-up. Also, obliteration was calculated from 69 SRS studies (GKRS-modality: 22/69) with a shorter median follow-up of 35 months. In our pooled analysis, 60.5% of patients achieved angiography-confirmed obliteration and overall nidus obliteration rate was 69.7% with a median time to obliteration of 35.4 months after initial radiosurgical intervention. Whilst realisation of nidus obliteration on neuroimaging occurs at relatively varying intervals due to differences in the intervals of radiological follow-up, our median study follow-up (60 months) is a sufficient duration, we believe, to accommodate this post-GKRS latency period whilst supporting the validity of our findings.

On behalf of the International Stereotactic Radiosurgery Society (ISRS), a systematic review was performed to establish SRS practice guidelines for SM I/II AVMs [30]. Of 1102 SM I/II AVMs (78% SM II, median margin dose 23 Gy, volume 2.4 cm^3^), overall reported obliteration rate was 80%. Of 888 SM I/II AVMs (72% SM II) in our study, 74% achieved obliteration. Both findings highlight that appropriately selected, low-grade AVMs can expect a significant chance of successful outcome.

Spetzler-Martin grade III AVMs are angioarchitecturally heterogeneous lesions, straddling the boundary between low grade (I–II) AVMs, which favour intervention with microsurgical resection, and high grade (IV–V) AVMs, which favour conservative management. Our findings suggest grade III AVMs experience a reasonable risk-to-benefit profile following GKRS; of 505 AVMs, 69% achieved obliteration.

Whilst our pooled and SM-stratified obliteration values entice direct comparisons with obliteration outcomes following surgical resection, caution is warranted as patient selection for GKRS versus surgical resection is undoubtedly to have been influenced by individual patient and AVM features which were not available for further detailed analysis.

Previous studies have reported on patient, AVM and treatment factors associated with the development of RICs, of which, increasing radiation dose and irradiated AVM volume, have been well established [5, 13, 17, 40]. Kano et al. investigated the incidence of and treatment parameters that contributed to the development of RICs following AVM GKRS. Further, 775 patients (median volume 3.6 cm^3^ and dose 20 Gy) underwent single-stage GKRS with ≥ 2 years of follow-up, with reported symptomatic and permanent RIC rates of 7.1% and 3%, respectively. Increased AVM volume, 12-Gy volume, higher margin dose and deep location were associated with a higher rate of developing symptomatic RICs [40]. In our meta-analysis, we found no significant association between either median study margin dose or AVM volume and the development of transient symptomatic (*p* = 0.45 and 0.43) or permanent RICs (*p* = 0.17 and 0.19). We believe that this may be due to lack of individual patient data, differences in patient selection and variations in GKRS treatment protocols among included studies; all of which precluded a sufficiently rigorous analysis of the relationship among study nidus volume, margin dose, and symptomatic RIC occurrence. Furthermore, this dose-volume relationship has been further refined to specify the ‘12-Gray volume’, the volume of brain tissue receiving radiation dose of 12 Gy or more, which is strongly correlated with the risk of RICs [20, 55]. Interestingly, only one study in our review [64] referred to the 12-Gy volume in their analysis of RIC outcomes. Studies suggest these dose-volume relationships are more strongly associated with the development of post-SRS RICs and less influential for the occurrence of symptomatic RICs [29, 55].

One AVM angioarchitectural feature consistently reported to be associated with the development of symptomatic RICs is deep location, more specifically, the brainstem [19, 21, 22, 61]. This was confirmed in our findings for both transient (*p* = 0.002) and permanent symptomatic RICs (*p* = 0.005) and is reflected in the RBAS grading system where deep location confers a higher risk feature associated with poorer outcome post-GKRS [61, 62].

The relative risk of AVM haemorrhage during the latency period between radiosurgery and obliteration versus an untreated AVM’s natural history remains a topic of debate [12]. In comparison to the generally accepted 2–4% natural haemorrhage rate of untreated AVMs [1], decreased, unchanged or increased rates of haemorrhage after radiosurgery have been reported [27, 50, 59]. In our pooled analysis, the annual haemorrhage rate following GKRS was 1.4%. This suggests radiosurgical intervention may afford partial protection from AVM rupture during the latency interval before nidus obliteration; however, we cannot definitively exclude that the decline in haemorrhage rate following GKRS is not part of the natural course of the disease or due to selection bias of included studies.

Further, in our analysis, we were unable to determine whether pre-GKRS AVM rupture status, yielded a significantly lower or higher post-GKRS haemorrhage risk as (1) the majority of studies did not stratify haemorrhage rate by rupture status, and (2) we did not have access to individual patient data to perform rigorous sub-group analysis within individual cohorts. Yen et al. [73] evaluated the rates of pre-and post-GKRS haemorrhage in a cohort of 1204 AVMs. The annual AVM haemorrhage risk from birth to radiosurgery, assuming patients are at risk of haemorrhage from birth, was 2.0% for the entire cohort and 3.7% for AVMs with prior haemorrhage. Post-GKRS, the annual haemorrhage risk until obliteration was 2.5%, stratified as 2.8% and 2.2% for AVMs with and without prior haemorrhage. Kano et al. [42] reported on a cohort of 407 ruptured AVMs. The annual haemorrhage rate between birth and radiosurgery was 3.4%, reducing to 1.3% following radiosurgical intervention. Overall, it appears GKRS reduces the haemorrhage risk of AVMs before obliteration, an attribute that may be more pronounced in patients who present with haemorrhage.

Cyst formation (CF) is an uncommon complication following SRS. In our review, 15 studies totalling 3446 patients reported a pooled incidence of 2%. This is approximated by the only comprehensive analysis of CF following GKRS by Ilyas et al., reporting a pooled CF rate of 2.9% in 2562 patients following GKRS, with a mean latency period of 6.5 years from time of GKRS to cyst detection [37]. A shorter median study follow-up (5 years) in our study, may partly explain a slightly reduced CF incidence. Reassuringly, in most cases, cysts are managed conservatively with relative success, especially if they are asymptomatic, radiologically stable or do not exert significant local mass effect [12].

### Limitations

The strengths of this review include adherence to PRISMA guidelines, its pre-specified protocol and formal validated risk-of-bias assessment. Studies included in this review generally followed a similar AVM GKRS treatment and follow-up protocol, of which the median length of follow-up across all studies was approximately 5 years.

In terms of limitations, the included studies were all non-randomised, mostly retrospective and at moderate risk of bias. In most analyses, heterogeneity was present and substantial, with the highest *I*^2^ values found in cohorts reporting obliteration outcomes. We are aware that certain studies may have been excluded whilst strictly adhering to our inclusion criteria which required outcomes data on obliteration, haemorrhage and RICs cumulatively or excluded solely due to English language restriction (Appendix 3, 4).

Most studies (29) in this review reported an aggregated angiography or MRI confirmed obliteration rate, some in addition or as an alternative to an angiography-only confirmed obliteration rate. Whilst MR-imaging has been shown to exhibit 77–85% sensitivity and 89–95% specificity for AVM obliteration detection [48, 63], there is a risk of incorrectly assuming AVMs to be obliterated by MRI at the time of assessment with subsequent over-estimation of the true nidus obliteration rate, especially when obliteration status is confirmed solely using MRI. False-positive nidal obliteration according to MRI can be detrimental to the patient because any residual arteriovenous shunting represents a persistent haemorrhage risk with the subsequent possibility of adverse outcome. In our pooled analysis, 60.5% of patients achieved angiography-confirmed obliteration whilst overall nidus obliteration rate, confirmed with either angiography or MRI, was 69.7%. Our findings for SM-stratified obliteration rates also illustrated a consistent and clinically significant incongruence between rates of obliteration confirmed by angiography or MRI and obliteration confirmed by angiography alone. Whilst it can be appreciated why some patients may choose to forgo DSA, for example due to its invasiveness, risk of procedural complications or false reassurance from the absence of AVM or GKRS-related symptoms, ultimately it remains the gold standard of accurately assessing AVM obliteration status and should remain the critical and definitive determinant of nidal obliteration in any post-GKRS imaging protocol [10, 33].

In this review, we obtained aggregate patient, AVM and outcome data with a tendency for studies to report on the whole patient cohort with limited further sub-group analysis. Very few studies stratified outcomes by individual patient and AVM features, e.g. by location, AVM volume, margin dose or SM grade, which have been well-established to affect radiosurgical outcomes. Further, the reporting of a clinically heterogeneous cohort of patients with AVMs is likely to contribute to heterogenous pooled estimates of outcomes, as illustrated in our findings. Interpreting these summary outcome estimates, without robust sub-group analysis can mitigate the generalisability of our findings for individual patients and future AVM treatment decision-making.

Standardised prospective multi-centre recording of patient, AVM and treatment characteristics and reporting of outcomes is needed to be certain of individual AVM risks and benefit prediction. Stratifying clinical outcomes further by AVM and pre-specified treatment subgroups will contribute to the selection of a more homogenous set of AVMs from which meaningful comparisons of outcomes can be made and valid conclusions can be drawn.

## Conclusion

Gamma knife radiosurgery is a safe and effective treatment option for cerebral AVMs. Appropriately selected patients can expect a significant chance of successful obliteration coupled with minimal risks of haemorrhage and radiation-induced complications. Future studies would be strengthened by attempting to report on a homogenous set of study participants, in terms of pre-specified patient, AVM angioarchitectural or treatment parameters, which would allow for a more conclusive risk: benefit profile of AVM GKRS to be established.

## Supplementary Information

Below is the link to the electronic supplementary material.

Supplementary file1 (PDF 764 kb)

## Data Availability

The analysis for this study is based on published results from individual studies. See Appendix; all extracted data from individual studies, code used for performing the meta-analyses and sensitivity analysis can be made available upon reasonable request to the corresponding author.

## Code availability

The code used for performing the meta-analyses and sensitivity analysis can be made available upon reasonable request to the corresponding author.
