# Supplementary material for: Gamma Knife radiosurgery for cerebral arteriovenous malformations: a systematic review and meta-analysis
Source: Neurosurg Rev. 2022 Feb 18;45(3):1987–2004. doi: 10.1007/s10143-022-01751-1 (PMC9160151; doi:10.1007/s10143-022-01751-1)

## APPENDIX 1. Checklists for reporting standards

***PRISMA Checklist***

| Section/topic | # | Checklist item | Reported |
|---|---|---|---|
| **TITLE** | | | |
| Title | 1 | Identification as a systematic review, meta-analysis, or both. | ✓ |
| **ABSTRACT** | | | |
| Structured summary | 2 | Structured abstract including background, objectives, data sources, study eligibility criteria, methodological assessment, synthesis method, results, conclusions and implications of key findings. | ✓ |
| **INTRODUCTION** | | | |
| Rationale | 3 | Describe the rationale for the review in the context of what is already known. | ✓ |
| Objectives | | Provide an explicit statement of questions being addressed with reference to participants, interventions, outcomes (PICO design). | ✓ |
| **METHODS** | | | |
| Protocol and registration | 4 | Indicate if a review protocol exists, and where it can be accessed. | ✓ |
| Eligibility criteria | 5 | Specify study characteristics and report characteristics (such as years considered, language, publication status) used as criteria for eligibility. | ✓ |
| Information sources | 6 | Describe all information sources (such as databases with dates of coverage, contact with study authors, experts) in the search, and the date of last search. | ✓ |
| Search | 7 | Present full electronic search strategy, including limits used, such that it could be repeated. | ✓ |
| Study selection | 8 | State the process for selecting studies (i.e., screening, eligibility) and make sure that this is done by 2 authors. | ✓ |
| Data collection | 9 | Describe method of data extraction from reports (e.g., piloted forms, independently, in duplicate) and any processes for obtaining and confirming data from investigators. | ✓ |
| Data items | 10 | List and define all variables for which data were sought (e.g., PICOS, funding sources) and any assumptions and simplifications made. | ✓ |
| Risk of bias in individual studies | 11 | Describe methods used for assessing risk of bias of individual studies (including specification of whether this was done at the study or outcome level), and how this information is to be used in any data synthesis. | ✓ |
| Summary measures | 12 | State the principal summary measures (e.g., risk ratio, difference in means). | ✓ |
| Synthesis of results | 13 | Describe the methods of handling data and combining results of studies, if done, including measures of consistency (e.g., $I^2$) for each meta-analysis. | ✓ |
| Risk of bias across studies | 14 | Specify any assessment of risk of bias that may affect the cumulative evidence (e.g., publication bias, selective reporting within studies). | ✓ |
| Additional analyses | 15 | Describe methods of additional analyses (e.g., sensitivity or subgroup analyses, meta-regression), if done, indicating which were pre-specified. | ✓ |
| **RESULTS** | | | |
| Study selection | 16 | Give numbers of studies screened, assessed for eligibility, and included in the review, with reasons for exclusions at each stage, illustrated with a flow diagram. | ✓ |
| Study characteristics | 18 | For each study, present characteristics for which data were extracted (e.g., study size, PICOS, follow-up period) and provide the citations. | ✓ |
| Risk of bias within studies | 19 | Present data on risk of bias of each study and, if available, any outcome level assessment. | ✓ |
| Results of individual studies | 20 | For all outcomes considered (benefits or harms), present, for each study: (a) simple summary data for each intervention group (b) effect estimates and confidence intervals, ideally with a forest plot. | ✓ |
| Synthesis of results | 21 | Present results of each meta-analysis done, including confidence intervals and measures of consistency. | ✓ |
| Risk of bias across studies | 22 | Present results of any assessment of risk of bias across studies. | ✓ |
| Additional analysis | 23 | Give results of additional analyses, if done (e.g., sensitivity or subgroup analyses, meta-regression). | ✓ |
| **DISCUSSION** | | | |
| Summary of evidence | 24 | Summarize the main findings including the strength of evidence for each main outcome; consider their relevance to key groups (e.g., healthcare providers, users, and policy makers). | ✓ |
| Limitations | 25 | Discuss limitations at study and outcome level (e.g., risk of bias), and at review-level (e.g., incomplete retrieval of identified research, reporting bias). | ✓ |
| Conclusions | 26 | Provide a general interpretation of the results in the context of other evidence, and implications for future research. | ✓ |
| **FUNDING** | | | |
| Funding | 27 | Describe sources of funding for the systematic review and other support (e.g., supply of data); role of funders for the systematic review. | ✓ |

## APPENDIX 2. Search strategies

**OVID Medline and Embase**

1. Gamma knife surgery/
2. Gamma knife/
3. GKRS/
4. GKS/
5. GK/
6. Radiosurg*/
7. Stereotactic
8. 1 or 2 or 3 or 4 or 5 or 6 or 7
9. Arteriovenous Malformation/
10. AVM
11. 9 or 10
12. 8 and 11
13. (brain* or cerebral or intracerebral or central nervous system or intracranial or cerebellar or intraventricular or supratentorial).tw.
14. 12 and 13 (English language only)

Number of references identified: 3402
Last date searched: October 17, 2021

**Open Grey**

1. arteriovenous malformation
2. AVM
3. radiosurgery

Number of references identified: 41
Last date searched: October 17, 2021

**Cochrane**

1. cerebral arteriovenous malformation or cerebral arteriovenous malformations or intracranial arteriovenous malformations or AVM
2. stereotactic or radiosurgery or gamma knife
3. 1 and 2

Number of references identified: 48
Last date searched: October 17, 2021

## APPENDIX 3. Excluded studies, with the reasons for their exclusion

| First author, year of publication | No access/Non-english/Not full paper | Study period pre- 1989 | No original data or review | <20 patients / < 1 year FU | No reporting of demographic data (Age/ Nidus volume/ Dose) | No reporting of location/SM/ Presentation | No reporting of clinical outcomes of interest (Obliteration/ Haemorrhage/ RICs) | LINAC/ Cyberknife/ Repeat GK/ Multi-stage GKRS | Superseded by another study |
|---|---|---|---|---|---|---|---|---|---|
| Abecassis et al. 2017 | | | | | *[1] | | | | |
| Anderson et al. 2020 | | | | | | | | * | |
| Back et al. 2008 | | | | | * | * | | | |
| Bowden et al 2014 “in/near Sylvian fissure” | | | | | | | | | *[2] |
| Bowden et al 2014 “near ventricles” | | | | | | | | | *[2] |
| Bowden et al. 2014 “cerebellum” | | | | | | | | | *[2] |
| Bowden et al. 2015 | | | | | | | | | *[2] |
| Bowden et al. 2017 | | | | | | | | | *[2] |
| Burrow et al. 2014 | | | | | | | | | *[3] |
| Chen, C.J et al. 2018 | | | | | | | | | *[4] |
| Chen, C.J et al. 2019 “seizures” | | | | | | | | | *[4] |
| Chen, C.J et al. 2019 “micro vs GK” | | | | | | | | | *[5] |
| Chen, C.J et al. 2020 | | | | | | | | | *[4] |
| Cheng C.H et al 2012 | | | | | | | | | *[5] |
| Coffey et al. 1995 | * | | | | | | | | |
| Cohen-inbar et al. 2015 | | | | | | | | | *[5] |
| Cohen-Inbar O. et al. 2017 “cerebellar” | | | | | | | | | *[4] |
| Cohen-Inbar O. et al. 2017 “early vs late” | | | | | | | | | *[4] |
| Da Costa et al. 2009 | | | | | | | * | | |
| Ding et al 2013 “eloquent locations” | | | | | | | | | *[5] |
| Ding et al 2014 “cerebellar” | | | | | | | | | *[5] |
| Ding et al. 2014 “Ruptured AVMS” | | | | | | | | | *[5] |
| Ding et al. 2015 “effect of prior haemorrhage on GK outcomes…” | | | | | | | | | *[5] |
| Ding et al. 2015 “temporal Lobe” | | | | | | | | | *[5] |
| Ding et al. 2016 “ARUBA eligible AVMs.” | | | | | | | | | *[4] |
| Ding et al. 2016 “repeat GK” | | | | | | | | | *[7] |
| Ding et al. 2017 “ARUBA Grade 1/2 AVMs” | | | | | | | | | *[4] |
| Ding et al. 2017 “Grade 3 AVMs” | | | | | | | | | *[4] |

| **First author, year of publication** | **No access/Non-english/Not full paper** | **Study period pre- 1989** | **No original data or review** | **<20 patients / < 1 year FU** | **No reporting of demographic data (Age/ Nidus volume/ Dose)** | **No reporting of location/SM/ Presentation** | **No reporting of clinical outcomes of interest** (Obliteration/ Haemorrhage/ RICs) | **LINAC/ Cyberknife/ Repeat GK/ Multi-stage GKRS** | **Superseded by another study** |
|---|---|---|---|---|---|---|---|---|---|
| Ding et al. 2017 “Unruptured AVMS” | | | | | | | | | *[4] |
| Ding et al. 2019 “Risk of… Hemorrhage” | | | | | | | | | *[4] |
| Douglas et al. 2008 | | | | | | * | | | |
| Duma et al. 1993 | * | | | | | | | | |
| El-Shehaby et al. 2019 | | | | | | | | * | |
| Flickinger et al. 1992 | | | | | | | * | | |
| Flickinger et al. 1997 | | | | | | | * | | |
| Flickinger et al. 1998 | | | | | | | * | | |
| Flickinger et al. 1999 | | * | | | | | | | |
| Flickinger et al. 2000 | | | | | | | * | | |
| Flickinger et al. 2002 | | | | | | | * | | |
| Franzin et al. 2016 | | | | | | | | * | |
| Friedman et al. 2003 | | | | | | | | * | |
| Graffeo et al. 2020 | | | | | | | | | *[3] |
| Hanakita et al. 2014 | | | | | | | | | *[6] |
| Hanakita et al. 2016 | | | | | | | | | *[6] |
| Hasegawa et al. 2017 | | | | | | | | | *[6] |
| Hasegawa et al. 2018 “a comprehensive study” | | | | | | | * | | *[6] |
| Hasegawa et al. 2018 adv age” | | | | | | | | | *[6] |
| Hasegawa et al. 2020 | | | | | | | | | *[6] |
| Hou et al. 1998 | * | | | | | | | | |
| Hung et al. 2020 | | | | | | | | | *[5] |
| Ilyas et al. 2017 | | | | * | | | | | |
| Inoue. 2006 | | | | | | * | | | |
| Izawa et al. 2009 | | | * | | | | | | |
| Jevalkar et al. 2009 | | | | * | | | | | |
| Jokura et al. 2009 | | | | | | * | | | |
| Kano et al. 2012 “Large” | | | | | | | | * | |
| Kano et al. 2017 “Estimating risks of ARE” | | | | | | | * | | |
| Kano et al. 2017 “Repeat -GK” | | | | | | | | * | |
| Karlsson et al. 1997 | | * | | | | | | | |
| Karlsson et al. 1999 | | * | | | | | | | |
| Karlsson et al. 2019 | | | | | * | | | | |

| First author, year of publication | No access/Non-english/Not full paper | Study period pre- 1989 | No original data or review | <20 patients / < 1 year FU | No reporting of demographic data (Age/ Nidus volume/ Dose) | No reporting of location/SM/ Presentation | No reporting of clinical outcomes of interest (Obliteration/ Haemorrhage/ RICs) | LINAC/ Cyberknife/ Repeat GK/ Multi-stage GKRS | Superseded by another study |
|---|---|---|---|---|---|---|---|---|---|
| Kasliwal et al. 2008 | | | | | | | * | | |
| Kawashima et al. 2020 | | | | | | | | | *[6] |
| Kemeny et al. 1989 | | | | * | | | | | |
| Kemeny et al. 2007 | | | | | * | | | | |
| Kihlstrom et al. 1997 | | | | * | | | | | |
| Kim H et al. 2010 | | | | | | | | * | |
| Kim M.J. et al. 2020 | | | | | | | * | | |
| Koga et al. 2010 | | | | | | | | | *[6] |
| Koga et al. 2012 | | | | | | | | | *[6] |
| Koltz et al. 2013 | | | | | * | | | | |
| Kurita et al. 2000 "occipital" | | | | | | | | | *[6] |
| Kurita et al. 2000 "brainstem" | | | | | | | | | |
| Lang et al.2018 | | | | | | | | | |
| Lenck et al. 2018 | | | | | | | | * | |
| Lunsford et al. 1989 | | | | | | | | | *[2] |
| Lunsford et al. 1990 | * | | | | | | | | |
| Lunsford et al. 1992 | * | | | | | | | | |
| Marciscano A.E. et al. 2017 | | | * | | | | | | |
| Maruyama et al. 2004 | | | | | | | | | *[2] |
| Maruyama et al. 2005 | | | | | | | | | *[6] |
| Maruyama et al. 2005 "corpus Callosum" | | | | | | | | | *[6] |
| Nagy et al. 2012 "deep" | | * | | | | | | | |
| Nagy et al. 2012 "large" | | * | | | | | | | |
| Nagy et al. 2017 | | | | | | | | * | |
| Nerva et al. 2015 | | | | | * | | | | |
| Nicolato et al. 2006[i] Part 1 | | | | | | | *[8] | | |
| Nicolato et al. 2006[i] Part 2 | | | | | | | *[8] | | |
| Niranjan et al. 2018 | | | | | | | | | *[2] |
| Park et al. 2016 | | | | | | | | * | |
| Patibandla M.R. et al. 2018 | | | | | | | | | *[4] |
| Patibandla M.R. et al. 2019 | | | | | | | | | *[4] |
| Paul et al. 2014 | | | | | | | * | | |
| Pollock et al. 1994 | * | | | | | | | | |
| Pollock et al. 1996 "factors that predict" | | | | | | | * | | |
| Pollock et al. 1996 "haemorrhage risk" | | | | | | | | | *[2] |

| **First author, year of publication** | **No access/Non-english/Not full paper** | **Study period pre- 1989** | **No original data or review** | **<20 patients / < 1 year FU** | **No reporting of demographic data (Age/ Nidus volume/ Dose)** | **No reporting of location/SM/ Presentation** | **No reporting of clinical outcomes of interest** (Obliteration/ Haemorrhage/ RICs) | **LINAC/ Cyberknife/ Repeat GK/ Multi-stage GKRS** | **Superseded by another study** |
|---|---|---|---|---|---|---|---|---|---|
| Pollock et al. 1996 “post-geniculate” | | | | | | | * | | |
| Pollock et al. 1998 | | | | | | | | | *[3] |
| Pollock et al. 1999 | | | | | * | | | | |
| Pollock et al. 2003 | | | | | | | | | *[3] |
| Pollock et al. 2004 | | | | | | | | | *[3] |
| Pollock et al. 2013 | | | | | | | | | *[3] |
| Pollock et al. 2017 | | | | | | | | * | |
| Pomeraniec et al. 2018 | | | | | | | | | *[5] |
| Potts M.B. 2014 | | | | | | | * | | |
| Safain M.G. et al. 2014 | | | | * | | | | | |
| Sasaki et al. 1998 | | * | | | | | | | |
| Schwyzer et al. 2012 | | | | | | | | | *[5] |
| Serizawa et al. 2012 | * | | | | | | | | |
| Seymour et al. 2020 | | | | | | | | * | |
| Shi et al. 2007 | * | | | | | | | | |
| Shin et al. 2004 | | | | | | | | | *[6] |
| Sirin et al. 2006 | | | | | | | | | *[2] |
| Starke et al. 2013 | | | | | | | | | *[5] |
| Starke et al. 2017 | | | | | | | | | *[4] |
| Steiner et al. 1992 | | * | | | | | | | |
| Sun et al. 2011 | | | | | | * | | | |
| Sung et al. 2009 | | | | | | | | * | |
| Tonetti et al. 2018 | | | | | * | | | | |
| Wegner et al. 2011 | | | | | | * | | | |
| Yamamoto et al. 1992 | | * | | | | | | | |
| Yamamoto et al. 1992 | | * | | | | | | | |
| Yamamoto et al. 1995 | | | | | | | | | *[3] |
| Yamamoto et al. 1996 | | * | | | | | | | |
| Yamamoto et al. 1998 | | * | | | | | | | |
| Yamamoto et al. 2012 | | | | | | | | * | |
| Yamamoto et al. 2013 | | * | | | | | | | |
| Yang et al. 2012 | | | | | | * | | | |
| Yen C.P et al. 2010 | | | | | | | | * | |
| Yen C.P et al. 2013 | | | * | | | | | | |
| Yen C.P et al. 2014 | | | | | | | | | *[5] |
| **Total number of studies:** | **8** | **12** | **3** | **5** | **8** | **7** | **16** | **15** | **57** |

[1]This study reported on the outcomes following multimodal management for SM III AVMs. However, it was not possible to extract demographic data (Age/Margin dose/Median Nidus volume) specifically for those patients treated with Gamma Knife. [2] Superseded by Kano et al 2012 [41, 43-45] [3]Superseded by Pollock *et al,* 2016 [64] [4]Superseded by Chen, C.J et al, 2018 [7] [5]Superseded by Ding et al. 2014 [14-16] [6] Superseded by Hasegawa et al. 2018 [32] [7] Superseded by Yen C.P *et al* 2011 [73]. [8]Nicolato et al. Part 1 and 2, 2006 were both excluded following senior discussion and review as both papers in combination do meet minimum outcomes dataset for inclusion, and it was not possible to assume that both studies were referring to the exact same patient set. The corresponding author was contacted via email for further data, however, a response is still pending at time of article submission.

# APPENDIX 4. Overview of overlapping studies

| Institution | First author and year of publication | Treatment Period | Number of patients | Median Follow-up (Months) | Included/ excluded with reason for exclusion |
|---|---|---|---|---|---|
| **University of Virginia** | Ding *et al* 2014 (SM 1/2) | 1989-2012 | 502 patients SM1/2 | Clinical: 61.6 (6.8–239.4) | Included. |
| | Ding *et al* 2014 (SM 3) | 1989-2009 | 398 patients SM3 | Clinical: 67.5 (6.2–239.2) | Included. |
| | Ding *et al* 2014 (SM 4/5) | 1989-2009 | 110 patients SM4/5 | Clinical: 97.4 (6.5– 312.9) | Included. |
| | Yen C.P *et al* 2009 Repeat GK cohort | 1989-2007 | 140 patients | Mean Clinical: 84.2 (15-220) | Included. |
| | Ding *et al.* 2015 "effect of prior haemorrhage on GK outcomes…" | 1989-2009 | 270 patients All Ruptured cohort | Mean Clinical: 85.7 | Excluded.<br><br>Reason: superseded by Ding et al. 2014 SM1-2/3/4-5 |
| | Ding et al. 2016 "repeat GK" | 1989-2013 | 84 patients Repeat GK cohort | Mean: 77.6 (±SD 47.6) | Excluded.<br><br>Reason: Longer study time period 1989-2013 however fewer (case matched) patients at 84 compared to Yen C.P et al 2009: |
| | Starke *et al.* 2013 A practical grading… | 1989-2009 | 1012 patients | Mean: 96 (24–240) | Excluded.<br><br>Reason: superseded by Ding et al. 2014 SM1-2/3/4-5 which stratifies by SM grade |
| | Pomeraniec et al. 2018 | 1989-2015 | 1159 patients | Mean: 72.1 | Excluded.<br><br>Reason: Data split according to cyst formation and Ding et al. 2014 SM1-2/3/4-5 stratifies by SM grade |
| | Cohen-inbar *et al.* 2015 "A quantitative analysis of .." | 1987-2012 | 105 patients | Mean Clinical: 53.8 | Excluded.<br><br>Reason: superseded by Ding et al. 2014 SM1-2/3/4-5 |
| | Hung *et al.* 2020 "pre-embolisation vs GK-only" | 1989-2012 | 35 patients GK alone cohort | Angiography: 65 (13–48) | Excluded.<br><br>Reason: superseded by Ding et al. 2014 SM1-2/3/4-5 |
| | Chen, C.J *et al.* 2019 Microsurgery vs GK | 2001-2013 | 59 patients GK alone cohort | Mean Clinical: 92.1 (±SD 58.3) | Excluded.<br><br>Reason: superseded by Ding et al. 2014 SM1-2/3/4-5 |
| | Ding *et al.* 2014 Ruptured AVMS | 1989-2009 | 465 patients All Ruptured cohort | Mean Clinical: 72.2 (6.2–312.9) | Excluded.<br><br>Reason: superseded by Ding et al. 2014 SM1-2/3/4-5. Not possible to extract data for all patients. |
| | Yen C.P *et al* 2014 | 1989-2009 | 31 patients All Incidental findings | Clinical: 51(24-196) | Excluded.<br><br>Reason: superseded by Ding et al. 2014 SM1-2/3/4-5 |
| | Schwyzer *et al.* 2012 | 1989-2008 | 729 patients GK alone cohort | Mean Clinical: 79.2 (6-324) | Excluded.<br><br>Reason: superseded by Ding et al. 2014 SM1-2/3/4-5 |
| | Cheng C.H *et al* 2012 "BG and Thalamus" | 1989-2007 | 182 patients Basal ganglia and thalamus | Clinical: 85.8 (24-266) | Excluded.<br><br>Reason: Only Basal Ganglia and Thalamus located AVMs Superseded by Ding et al. 2014 SM1-2/3/4-5 |

| Institution | First author and year of publication | Treatment Period | Number of patients | Median Follow-up (Months) | Included/ excluded with reason for exclusion |
|---|---|---|---|---|---|
| | Ding *et al.* 2015 "temporal Lobe" | 1989-2012 | 175 patients Temporal lobe | Clinical 73.3 (6.2–230.4) | Excluded.<br><br>Reason: Only Temporal located AVMs Superseded by Ding et al. 2014 SM1-2/3/4-5 |
| | Ding *et al* 2014 "cerebellar" | 1989-2010 | 60 patients Cerebellar | Clinical: 52.8months (6.8 to 195.4) | Excluded.<br><br>Reason: Only Cerebellar located AVMs superseded by Ding et al. 2014 SM1-2/3/4-5 |
| | Ding *et al* 2013 "eloquent locations" | 1989-2009 | 134 patients Eloquent | 79.8 (8.4 to 223.5) | Excluded.<br><br>Reason: Only eloquent area located AVMs superseded by Ding et al. 2014 SM1-2/3/4-5 |
| **University of Pittsburgh** | Kano *et al* 2012 (SM1/2) | 1987-2006 | 217 patients SM1/2 | 64 (6–267) | Included. |
| | Kano *et al* 2014 (Grade 3) | 1987-2009 | 474 patients SM3 | 89 (2–278) | Included. |
| | Kano *et al* 2012 Part 4 (BG and thalamus) | 1987-2006 | 140 patients Basal Ganglia or Thalamus | Mean Clinical 81(2–265) | Included. |
| | Kano *et al* 2012 Part 5 (brainstem) | 1987-2006 | 67 patients Brainstem | 73.3 months (6–269) | Included. |
| | Kano *et al* 2012 Part 3 (repeat) | 1987-2006 | 105 patients Repeat-GK | 80 (6–205) | Included. |
| | Kano *et al* 2012 Part 6 (large multistage) | 1987-2006 | 47 patients Large 10cm3+ | 87 (0.4–206) | Included. |
| | Bowden *et al* 2014 "in/near Sylvian fissure" | 1987-2009 | 87 patients In/Near Sylvian fissure | 59 (6–295) | Excluded.<br><br>Reason: Only AVMs located in/near Sylvian fissure Superseded by Kano *et al. (include reference numbers)* |
| | Bowden *et al* 2015 "post-geniculate pathway" | 1987-2009 | 171 patients Along course of optic radiation | 74 (5–297) | Excluded.<br><br>Reason: Only AVMs located along optic radiation Superseded by Kano *et al. (include reference numbers)* |
| | Bowden *et al*. 2014 "cerebellum" | 1987-2007 | 64 patients Cerebellum | Radiological 73 (4–255) | Excluded.<br><br>Reason: Only Cerebellar located AVMs Superseded by Kano *et al. (include reference numbers)* |
| | Bowden *et al* 2014 "near ventricles" | 1987-2009 | 188 patients Within or in contact ventricles | Radiological 65 (2–265) | Excluded.<br><br>Reason: Only AVMs located in/near ventricles Superseded by Kano *et al. (include reference numbers)* |
| | Maruyama *et al* 2004 "brainstem" | 1987-2002 | 50 patients Brainstem | 56 (5-176) | Excluded.<br><br>Reason: Only Brainstem located AVMs Superseded by Kano *et al* 2012 Part 5 (brainstem) |
| | Niranjan *et al* 2018 "seizure presentation" | 1987-2012 | 28 patients Large volume-staged | 59 (6–295) | Excluded.<br><br>Reason: Only AVMs with seizure presentation Superseded by Kano *et al. large (include reference numbers)* |

| Institution | First author and year of publication | Treatment Period | Number of patients | Median Follow-up (Months) | Included/ excluded with reason for exclusion |
|---|---|---|---|---|---|
| | Bowden *et al* 2017 "impact on headaches" | 1995-2013 | 102 patients | 89.7 (13-249) | Excluded. Reason: Only AVMs with headache presentation Superseded by Kano *et al. (include reference numbers)* |
| | Pollock *et al*. 1996 haemorrhage risk… | 1987-1992 | 315 patients | Mean: 47 | Excluded. Reason: Superseded by Kano *et al. (include reference numbers)* |
| | Sirin *et al* 2006 Large | 1987-2004 | 28 patients Large volume-staged | 50 (3–15) | Excluded. Reason: Superseded by Kano *et al* 2012 Part 6 (large multistage) |
| | Lunsford et al. 1989 | 1987-1989 | 113 AVM cohort | not stated "minimum 6 months" | Excluded. Reason: Superseded by Kano *et al. (include reference numbers)* |
| **Mayo Clinic Rochester, USA** | Pollock *et al* 2016 "The effect of treatment periods…" | 1990-2009 | 371 patients (1990-1999 + 1999-2009 cohort) | 93 (3-290) | Included |
| | Pollock *et al 2017* "volume staged" large | 1997-2012 | 34 patients Large Volume-staged | 98.4 (36-159.6) | Included |
| | Graffeo *et al*. 2020 | 1990-2011 | 173 patients SM1/2 | 68 (24-275) | Excluded. Reason: Fewer case numbers and so superseded by Pollock *et al* 2016 *(include reference numbers)* |
| | Pollock *et al* 2003 patient outcomes after… | 1990-1997 | 144 patients | Mean: 86 (23–169) | Excluded. Reason: Superseded by Pollock *et al* 2016 *(include reference numbers)* |
| | Pollock *et al* 2013 risk of stroke… | 1990-2005 | 174 patients | 64 (IQR 36–120) | Excluded. Reason: Superseded by Pollock *et al* 2016 *(include reference numbers)* |
| | Pollock *et al* 2004 "BG thalamus, and BS" | 1990-2000 | 56 patients Deep located AVMs | 45 (12–121) | Excluded. Reason: Superseded by Pollock *et al* 2016 *(include reference numbers)* |
| | Pollock *et al.* 1998 "factors associated" | 1987-1992 | 220 patients | mean: 47 (±SD 20) | Excluded. Reason: No overlap 1987-1990 however fewer patients (220) compared to Pollock et al. 2016 (371) |
| | Yamamoto *et al* 1995 | 1990-1993 | 121 patients | 97 (54-205) | Excluded. Reason: Superseded by Pollock *et al* 2016 *(include reference numbers)* |
| | Burrow *et al* 2014 | 1990-2009 | 80 patients | Mean: 68 (12-133) | Excluded. Reason: Superseded by Pollock *et al* 2016 *(include reference numbers)* |

| Institution | First author and year of publication | Treatment Period | Number of patients | Median Follow-up (Months) | Included/ excluded with reason for exclusion |
|---|---|---|---|---|---|
| **University of Tokyo Hospital** | Hasegawa et al. 2018 “Comparison of the long term..” | 1990-2014 | 592 Patients | 92 (1–320) | Included. |
| | Hanakita *et al.* 2016 ARUBA eligible | 1990-2010 | 292 Patients ARUBA-eligible | 62 (IQR 36–106) | Excluded.<br><br>Reason: Superseded by Hasegawa *et al.* 2018 which includes ARUBA eligible cohort. |
| | Hanakita *et al.* 2014 | 1998-2010 | 67 patients Large (10cm3+) | 55 (7-178) | Excluded.<br><br>Reason: Superseded by Hasegawa *et al.* 2018 which includes large AVMs |
| | Maruyama *et al.* 2005 | 1990-2003 | 500 patients | 93.6 (IQR 115.2) | Excluded.<br><br>Reason: Superseded by Hasegawa *et al.* 2018 (include reference number) |
| | Shin *et al.* 2004 | 1990-1999 | 408 patients | 65 (1-135) | Excluded.<br><br>Reason: Superseded by Hasegawa *et al.* 2018 |
| | Kawashima *et al.* 2020 “Ruptured AVMs” | 1990-2016 | 410 patients All Ruptured cohort | 111 (1–351) | Excluded.<br><br>Reason: Only ruptured AVMs Superseded by Hasegawa *et al.* 2018 |
| | Kurita *et al* 2000 “occipital” | 1990-1997 | 37 patients Occipital lobe | 46 12-103 | Excluded.<br><br>Reason: Only Occipital lobe located AVMs Superseded by Hasegawa *et al.* 2018 |
| | Kurita *et al* 2000 “brainstem” | 1990-1997 | 30 patients Brainstem | Mean: 52.2 (±SD 31.3) | Excluded.<br><br>Reason: Only brainstem located AVMs Superseded by Hasegawa *et al.* 2018 |
| | Hasegawa *et al. 2017* “cerebellar” | 1990-2010 | 45 patients Cerebellum | 120 (5-291) | Excluded.<br><br>Reason: Only cerebellar located AVMs Superseded by Hasegawa *et al.* 2018 |
| | Hasegawa et al. 2020 “re-evaluating” | 1990-2014 | 791 patients (Adults+Paediatrics) | 83 (1-320) | Excluded.<br><br>Reason: Superseded by Hasegawa *et al.* 2018 which excludes paediatric patients. |
| | Hasegawa et al. 2018 “a comprehensive study” | 1990-2010 | 581 patients | 121.2 (24-320.4) | Excluded.<br><br>Reason: No data on RICs |
| | Hasegawa et al. 2018 adv age” | 1990-2013 | 561 Patients Age 65 years + versus 65- | 91 | Excluded.<br><br>Reason: Superseded by Hasegawa *et al.* 2018 |
| | Maruyama *et al* 2005“corpus Callosum” | 1990-2002 | 32 patients Corpus Callosum | 108 (12-144) | Excluded.<br><br>Reason: Only corpus callosum located AVMs Superseded by Hasegawa *et al.* 2018 |

| Institution | First author and year of publication | Treatment Period | Number of patients | Median Follow-up (Months) | Included/ excluded with reason for exclusion |
|---|---|---|---|---|---|
| | Koga *et al* 2010 "SRS thalamus" | 1990-2009 | 48 patients Thalamus | 45 (6-198) | Excluded.<br><br>Reason: Only Thalamus located AVMs Superseded by Hasegawa *et al.* 2018 |
| | Koga et al. 2012 "tractography" | Not Stated ("from 2000") | 52 patients Diffusion Tensor Tractography treatment planning cohort | 48 (36-80) | Excluded.<br><br>Reason: Superseded by Hasegawa *et al.* 2018 which includes DTT cohort |
| **Multi-Center (International Gamma Knife Research Foundation)**:<br>USA: University of Virginia, University of Pittsburgh, University of Louisville, Cleveland Clinic Foundation, New York University, Beaumont Health System, Michigan<br>Canada: University of Sherbrooke, Quebec.<br>Puerto Rico: University of Puerto Rico, San Juan. | Chen, C.J *et al. 2018* (paediatric vs adult AVM) | 1987-2014 | 1845 patients Adults | Mean: 80.7 (±SD 62.5) | Included. |
| | Seymour *et al.* 2020 "volume staged GK IGKRF" | 1991-2016 | 257 patients Two-stage+ | 69.5 (1.68-302.4) | Included. |
| | Starke *et al.* 2017 | 1988-2013 | 2236 patients Adult+Paediatrics | Mean: 84 (6-240) | Excluded.<br><br>Reason: Unlike Chen *et al. 2018 does not split data (same) into adult:Paediatric* |
| | Ding *et al.* 2019 "Risk of… Hemorrhage" | 1987-2014 | 2320 patients | Mean: 80.36 (±SD 62.4) | Excluded.<br><br>Reason: No data on RICs which were included in Chen *et al. 2018* |
| | Patibandla *et al.* 2019 "—> "outcomes pre/post-2000" | 2001–2014 | 664 patients | Mean: 45.0 ± 29.6 | Excluded.<br><br>Reason: Superseded by Chen *et al. 2018* which includes 1987-2000 data |
| | Chen, C.J *et al.* 2018 "Advanced age…" | 1987-2014 | 1845 patients Adults | Mean: 80.7 (±SD 62.5) | Excluded.<br><br>Reason: Same dataset as Chen C.J *et al. 2018 but splits data into age 65 +/-* |
| | Chen, C.J *et al.* 2020 "Basal ganglia and thalamus" | 1987-2014 | 363 patients Basal Ganglia or Thalamus | Mean: 86.5 | Excluded.<br><br>Reason: Same data as Chen *et al. 2018 but only includes patients with Basal ganglia or Thalamus located AVMs* |
| | Chen, C.J *et al.* 2019 "seizures" | 1987-2014 | 419 patients | Mean: 37.2 (±SD 16) | Excluded.<br><br>Reason: Unlike Chen *et al. 2018 does not split data (same) into adult:Paediatric* |
| | Cohen-Inbar *et al.* 2017 | 1988-2014 | 1398 patients All obliterated | | Excluded.<br><br>Reason: Outcome data only on patients who had proven obliteration |
| | Ding *et al.* 2017 ARUBA-eligible Grade 1/2 AVMs | 1987-2014. | 232 patients SM1/2 | Mean: 90.5 (12.0-324.7) | Excluded.<br><br>Reason: Only SM1/2 ARUBA-eligible AVMs Superseded by Chen *et al. 2018* |

| Institution | First author and year of publication | Treatment Period | Number of patients | Median Follow-up (Months) | Included/ excluded with reason for exclusion |
|---|---|---|---|---|---|
| | Ding *et al.* 2017 Grade 3 AVMs | 1987-2014 | 891 patients SM3 | Mean: 88.6 (12.0 to 278.4) | Excluded. Reason: Only SM3 ARUBA-eligible AVMs Superseded by Chen *et al. 2018* |
| | Ding *et al.* 2016 ARUBA eligible AVMs. | 1987-2014. | 509 patients ARUBA-eligible | Mean: 86.2 (±SD 62.3) | Excluded. Reason: Only ARUBA-eligible AVMs Superseded by Chen *et al. 2018* |
| | Ding *et al.* 2017 Unruptured AVMS | Not stated | 938 patients All Unruptured | 70.9 (12.0-275.6) | Excluded. Reason: Only unruptured AVMs Superseded by Chen *et al. 2018* |
| | Patibandla M.R. *et al.* 2018 Grade 4/5 | Not stated | 233 patients (SM4/5) | 84.5 (±SD 59.5) | Excluded. Reason: Only SM4/5 AVMs Superseded by Chen *et al. 2018* |
| | Cohen-Inbar O. *et al.* 2017 | 1988-2015 | 162 patients Cerebellar | Median 60 (7–325) | Excluded. Reason: Only Cerebellar AVMs Superseded by Chen *et al. 2018* |

[a]Median clinical or radiological follow-up, whichever is longer. [b]ARUBA (A Randomised **trial** of Unruptured Brain Arteriovenous malformation.

# APPENDIX 5. Risk of bias Summary ROBINS-I

| First author, year of publication | Bias due to confounding | Bias in selection of participants into the study, selection bias | Bias in classification of interventions | Bias due to deviation from intended interventions, performance bias | Bias due to missing data, <80%, attrition bias | Bias in measurement of outcomes, detection bias | Bias in selection of the reported result, outcome reporting bias |
|---|---|---|---|---|---|---|---|
| Arslan *et al.* 2017 [2] | Yellow | Yellow | Green | Green | Yellow | Yellow | Green |
| Bir *et al.* 2015 [4] | Yellow | Green | Green | Green | Green | Yellow | Green |
| Bose *et al.* 2015 [5] | Yellow | Green | Green | Green | Green | Yellow | Green |
| Chang *et al.* 2000 [6] | Yellow | Green | Green | Green | Yellow | Yellow | Yellow |
| Chen *et al.* 2018 [7] | Yellow | Yellow | Green | Green | Green | Green | Green |
| Choe *et al.* 2008 [8] | Yellow | Green | Green | Green | Yellow [a] | Green | Green |
| Ding *et al.* 2014 [14] | Yellow | Yellow | Green | Green | Green | Green | Green |
| Ding *et al.* 2014 [15] | Yellow | Yellow | Green | Green | Green | Green | Green |
| Ding *et al.* 2014 [16] | Yellow | Yellow | Green | Green | Green | Green | Green |
| Ditty *et al.* 2017 [18] | Yellow | Green | Green | Green | Green | Yellow | Green |
| Franzin *et al.* 2013 [24] | Yellow | Green | Green | Green | Yellow | Yellow | Green |
| Han *et al.* 2008 [31] | Yellow | Yellow | Green | Green | Yellow | Yellow | Green |
| Hasegawa *et al.* 2018 [32] | Yellow | Yellow | Green | Green | Green | Green | Green |
| Hirschmann *et al.* 2019 [35] | Yellow | Green | Green | Green | Yellow | Green | Green |
| Hu *et al.* 2020 [36] | Yellow | Yellow | Green | Green | Green | Yellow | Green |
| Izawa *et al.* 2005 [39] | Yellow | Yellow | Green | Green | Green | Green | Green |
| Kano *et al.* 2014 [41] | Yellow | Green | Green | Green | Green | Yellow | Green |
| Kano *et al.* 2012 [43] | Yellow | Green | Green | Green | Green | Yellow | Green |
| Kano *et al.* 2012 [44] | Yellow | Green | Green | Green | Green | Yellow | Green |
| Kano *et al.* 2012 [45] | Yellow | Yellow | Green | Green | Green | Yellow | Green |
| Kim BS *et al.* 2019 [46] | Yellow | Yellow | Green | Green | Green | Yellow | Green |
| Kiran *et al.* 2009 [47] | Yellow | Yellow | Green | Green | Yellow | Green | Green |
| Liscak *et al.* 2007 [49] | Yellow | Green | Green | Green | Yellow | Green | Yellow |
| Matsunaga et Shuto. 2014 [51] | Yellow | Green | Green | Green | Green | Yellow | Green |
| Missios *et al.* 2014 [52] | Yellow | Yellow | Green | Green | Green | Green | Green |
| Nicolato et al. 2002 [54] | Yellow | Green | Green | Green | Yellow | Green | Green |
| Orio *et al.* 2006 [56] | Yellow | Green | Green | Green | Yellow | Yellow | Yellow |
| Pan *et al.* 2000 [57] | Yellow | Green | Green | Green | Yellow | Green | Green |
| Parkhutik *et al.* 2013 [58] | Yellow | Yellow | Green | Green | Green | Green | Green |
| Pollock *et al.* 2016 [64] | Yellow | Yellow | Green | Green | Green | Yellow | Green |
| Raboud *et al.* 2018 [65] | Yellow | Green | Green | Green | Green | Yellow | Green |
| Tuleasca *et al.* 2020 [71] | Yellow | Green | Green | Green | Green | Green | Green |
| Zeiler *et al.* 2011 [74] | Yellow | Yellow | Green | Green | Green | Yellow | Green |
| Zhao *et al.* 2008 [75] | Yellow | Green | Green | Green | Green | Green | Green |

Green indicates low risk of bias; yellow indicates moderate risk of bias; red indicates serious risk of bias; grey indicates no information on which to base a judgement about risk of bias for this domain [a]Choe et al. 2008 [8] has missing radiological follow-up data (MRI) for 24 patients (/100) affecting obliteration & RICs data which has been attributed to '*incomplete short term follow up*' and additionally, '*a signif[i]cant proportion of lost patients before reaching final outcome*'. [b] Raboud et al. 2018 [65] is the single prospective study of all included studies (which are retrospective).

# APPENDIX 6. Detailed characteristics of included studies

| Study, year of publication | Total number of patients | Female patients (%) | Median age (range/ SD) | Location[2] (%) | Eloquent Location (%) | SM grade (%) | Venous Drainage (%) | Median Maximum Nidus Diameter, cm (range) | Associated flow-related or intranidal aneurysm (%) | Median RBAS OR VRAS Score | Pre-GKRS intervention (%) | Presentation with Haemorrhage (%) | Presentation with seizure (%) | Asymptomatic presentation (%) | Median Nidus Volume $cm^3$ (range) | Median Prescription dose Gy (Range) | Median Duration of follow-up months(range) | Number of patients with follow-up | Complete Obliteration rate — Angiography-confirmed (%) | Complete Obliteration rate — Angiography or MRI confirmed (%) | Median Time to obliteration (months) | Method of determining obliteration ± no. of patients underwent imaging modality | Post-GKRS Haemorrhage (%) | Post-GKRS transient RIC (radiological or symptomatic) | Post-GKRS permanent RIC (radiological or permanent FND) | Post-GKRS new onset/ increase frequency seizures | Cyst formation (%) | Post-GKRS Mortality (%) | Patients who underwent repeat-GKRS (%) | Outcomes for repeat-GK cohort |
|---|---|---|---|---|---|---|---|---|---|---|---|---|---|---|---|---|---|---|---|---|---|---|---|---|---|---|---|---|---|---|
| Arslan *et al.* 2017 [2] | 199 | 89 (45%) | 32 (3-74) | Lobar: 150 (75.4) Deep 35 ( 17.6) -Basal ganglia 10 (5) -Thalamus 10 (5) -Brainstem 15 (8) Corpus callosum 5 (3) Cerebellar 5 (5%) | — | I 16 (8.04) II 45 (22.61) III 119 (59.8) IV 14 (7.04) V 5 (2.51) | — | — | 7 (3.51) | 1.07 (0.18 – 4.80) | Embolisation 22 (11%) Surgical Resection 20 (10) Shunt 4 (2) EVD 3 (2) Ventriculostomy 1 (0.5) Embolisation + RS 1 (0.5) Surgery + RS 1 (0.5) Surgery + Embolisation 1 (0.5) | 86 (43) | 42 (21) | 1 (0.5) | 2.50 (0.05-39) | 22 (10-26 Gy) | 60.2 (7–100.1) | 199 | — | 141 /199 (71) | — | MRI AND Angiography: 153 (77%) MRI only: 46 (23%) | 7 (3.5%) | Total 54 (27%) including symptomatic in 13 (6.5%) | 0 (0) | 7 (3.5) | — | 4 (1.5). 3/4 ICH 1/4 Unrelated | 29 (14.6) | 10/29 obliteration |
| Bir *et al.* 2015 [4] | 85 | 42 (49.5) | 41 (17-76) | Cortical 68 (80) Deep 9 (10.6) -Thalamus 4/85 -Brain stem 3/85 -Basal ganglia 2/85 Cerebellum 9 (10.6) Corpus callosum 2/85 (2.35) | — | I 8 (9.41%) II 29 (34.1) III 25 (29.4%) IV 21 (24.71%) V 2 (2.4%) | — | 3 (0.4–9.5) | — | — | Embolisation 8 (9.4%) | 26 (30.5) | — | — | 3 (0.4-9.5) | Mean: 18 (14–25) | 32.65 (6–133) | 85 | | 67/85 (79) | 31 (5–89) | MRI AND Angiography: 30 (35%) MRI: 85 (100) | 5 (5.8%). annual rate 1.6%. | 0 (0) | 0 (0) | 0 (0) | 1 (1.2) | Total: 3 (3.56). 2/3 ICH 1/3 Hydrocephalus | 17 (20) | — |
| Bose *et al.* 2015 [5] | 96 | 34 (35.42) | Mean: 27.52 (±SD 14.48) | — | — | I 9 (9.38) II 31 (32.29) III 44(45.83) IV 11(11.46) V 1 (1.04) | — | — | — | — | Embolisation 23 (23.96) Surgical resection 4 (4.1) Gamma knife 10 (10.42) | 57 (59.4) | 0 (0) | — | Mean: 7.45 (1.01-71.2) | Mean 24.6 (15-25) | 24.5 (6-48) | 96 | 39 (72.2) | — | — | Angiography: 50 | 10 (10.42%) | Total transient or permanent symptomatic : 25 (26.04%) | Total transient or permanent symptomatic : 25 (26.04%) | — | — | Total: 2 (2.08%). ICH 2/2 | — | — |
| Chang *et al.* 2000 [6] | 278 | 117 (42) | Mean: 29.4 (2.8–68.1) | Lobar 156 (56.1%) [e]Deep 62 (22.3%) Corpus Callosum 17(6.1%) Cerebellum 23 (8.3%) Cerebrospinal fluid pathways 20 (7.2%) | — | I 37 (14.1%) II 88 (33.6%) III 84 (32.1%) IV 50 (19.1%) V 3(1.2%) | Deep 155 (59.2) Superficial 107(40.8) | 2.56 | 63 (26.3) | — | Embolisation 72 (25.9%) Surgical resection 20 (7.2) GK 23 (8.3%) | 179 (64.6) | 61 (21.9) | 7 (2.5) | 12.1 ( 0.15–109.5) | mean: 16.2 (8.4–30) | 24.7 (0.4–90.2) | 128 with radiological FU | /128 with 2 year`+ follow-up: 101 (78.9%) 101/128 AVMs Obliteration rate at 2 years/3/4 post-GKRS was 42%/ 56.7/78.2 | /128 with 2 year`+ follow-up: 101 (78.9%) 101/128 AVMs Obliteration rate at 2 years/3/4 post-GK was 42%/ 56.7/78.2 | — | Angiography | 19 (6.8%) | symptomatic 4(4.68%) | symptomatic 1(0.04%) | 8 (2.9) | 1 (0.4) | — | 23 (9.1) | — |
| Chen *et al.* 2018 [7] | 1845 | 929 (50.4%) | 40.4 (±SD 14.1) | Deep 384 (20.9%) | 1292 (70%) | I 209 (11.3%) II 699 (37.9%) III 751 (40.7%) IV 168 (9.1%) V 18 (1%) | Deep 1033 (56) Superficial 811 (44) | Mean: 2.4 (±SD 1.2) | 229/1845 (12.4%) | Mean: 1.4 (±SD 0.6) | Embolisation: 387 (21.3%) Surgical resection: 80 (4.4%) EBRT: 138 (7.5%) | 999 (54.2) | — | — | MEAN 4.6 (±SD 5.3) | Mean: 20.4 (±SD 3.8) | Mean: 80.7 (±SD 62.5) | 1845 | 896/1845 (48.6) | 1147/1845 (62.2) Actuarial obliteration rates at 2/4/6/8/10 years were 13.7%/38.8/ 47.2/53.5/ 57.1 | — | Angiography and MRI | 147 (8%) | Total: 545 (29.6%) including symptomatic 130 (7.04%) | symptomatic 42 (2.28%) | — | — | Total: 96 (7.9) all-causes | — | — |
| Choe *et al.* 2008 [8] | 100 | 48 (48) | Mean: 34 (5-66) | Cortical 67 (67%) Deep 18 (18) Cerebellum 11 (11) Corpus callosum 4 (4) | — | I 18 (18%) II 27 (27) III 36 (36) IV 11 (11) V 8 (8) | — | — | — | — | Embolisation 5 (5%) Surgical resection 2 (2) LINAC 3 (3) | 34 (34) | 25 (25) | 8 (8) | mean: 4.3(0.1 -29.3) | 20.8 (13-32) | Mean: 37.5 (5-63) | 100 | 28 /48 (58) Min. 3 year angiography follow-up: 29/35 (83) | — | Mean: 25.3 (6-43) | Angiography: 48 patients | 7 (7%). Annual rate 3.2% | Total /76 patients with MRI FU: Total: 24 (32%) including symptomatic 5 (6.6%) | 0 (0) | — | — | Total: 1 (1). 1/1 ICH | 16 (16) | — |
| Ding *et al.* 2014 [14] | 110 | 53 (48.2%) | 27.6 (4.7– 75.1) Age <18: 28 (25.5%) | Superficial 62 (56.4%) Deep 48 (43.6%) | 110 (100%) | IV 109 (99.1%) V 1 (0.9%) | Deep 110 (100) | 3.4 | — | 1.38 (0.47 – 4.10) | Embolisation 32 (29.1%) Surgical Resection 16 (14.5%). | 56 (50.9) | 23 (23) | 4 (3.6) | 5.7 (1.2 – 33.0) | 19 (10 – 25) | Clinical: 97.4 (6.5– 312.9) Radiological: 87.8 (17.3 – 261.6) | | 37/110 (33.6) | 48/110 (43.64) Actuarial obliteration rates at 3/5years were and 5 years were 10/23% | 42.7 (6.2–223.5) | Angiography and MRI | 20 (18.2%) Annual hemorrhage rate 3.0%. (800 risk years) | Total: 49 (48.0%) including symptomatic 10 (9.1%) | symptomatic 3 (2.73%) | 1 (1.3) | — | — | 34 (30.9) | — |
| Ding *et al.* 2014 [15] | 398 | 194 (48.7) | 30.9 (3.7–81.1) Age <18 yrs: 75 (18.8%) | Superficial 214 (53.8) Deep: 184 (46.2%) | 363 (91.2) | III 398 (100) | Deep 337 (84.7%) Multiple 169 (42.5%) | 2.3 (0.4–5.5) | — | 1.18 (0.21–3.70) | Embolisation 109 (27.4%) Surgical resection 44 (11.1%) | 234 (58.8) | 80 (20.1) | 11 (2.8) | 2.8 (0.1–27.8) | 20 (5–32) | Clinical: 67.5 (6.2–239.2) Radiological: 54.3 (5.3–230.4) | | 222/398 (55.8) | 276/398 (69.3) Actuarial obliteration rates: 3 and 5 years 38% and 60%, | 45.5 months | Angiography and MRI | 28 (7.03%). Annual hemorrhage rate 1.7%. (1897 risk-years) | Total /334 with 2 years+ follow-up: 115/334 (34.3%) including symptomatic 21/334 (6.3) | symptomatic 13/334 (3.9%) | 7 (1.8) | — | Total: Two (0.5). 2/2 ICH | 64 (16.1) | — |
| Ding *et al.* 2014 [16] | 502 | 249 (49.6%) | 35.2 (4.1-81.8) Age <18 yrs: 79 (15.7%) | Superficial 470 (93.6%) Deep 32 (6.4%) | 204 (40.6%) | I 147 (29.3%) II 355 (70.7%) | Deep 111 (22.1) Superficial 391 (77.9) | 2 (0.2–4.5) | — | 1.03 (0.21–2.95) | Embolisation: 101 (20.1%) Surgical Resection: 55 (11.0%). | 235 (46.8) | 126 (25.1) | 15 (3.0) | 2.4 (0.1–22.5) | 23 (7–36) | Clinical: 61.6 (6.8–239.4) Radiological: 47.9 (5.7–239.4) | | 304/502 (60.6) | 382/502 (76.1) Actuarial obliteration rates: 3/5/7/10 are 41/66/75/80% | 39.5 (5.7–192.8) | Angiography and MRI | 28 (5.6%) Annual hemorrhage rate 1.4% (2158 risk-years) | Total /414 patients with 2 year+ Follow-up: 160 (38.6%) including symptomatic in 28 (6.8%) | Total /414 patients with 2 year+ FU: 6 symptomatic (1.45%) | 15 (3.0) | 6 (1.2) | — | 50 (10.0) | — |
| Ditty *et al.* 2017 [18] | 78 | 39 (50) | Mean: 38.5 (6.9–74.3 years) | Cortical 69 (89.6%) Cerebellar 1(1.3%) Deep 6 (7.8) -Basal Ganglia 4/78 -Thalamus 1/78 -Brainstem 1/78 Ventricular 2 (2.6) | — | I 5 (6.5%) II 18 (23.4%), III 36(46.8%), IV 17 (22.1%) V 1(1.3%) | — | — | — | — | Embolisation 0 (0) Surgical resection 5 (6.4%) | 9 (14.3) | 78 (100) | — | — | 17.5 (17–22 Gy) | Mean: 36.4 (1–242.6) | 78 | — | /59 who underwent MRI or CTA or DSA: 34/59 (57.6) | — | MRI or CTA or DSA: 59 (75.6%) | 6 (7.69%) | symptomatic (transient or permanent): 6 (7.69%) | Radiation necrosis 3 (3.85%) | 5 (3) | — | — | 10 (12.8) | |
| Franzin *et al.* 2013 [24] | 127 | 56 (44.1) | 38.5 (6–76) | Lobar 106 (83.5) Deep 21 (16.5) | 80 (63%) | I 21 (16.5%) II 46 (36.2%) III 51 (40.1%) IV/V 9 (7.1%) | Deep 70 (55.1) Superficial 57 (44.9) | 1.8 | — | 1.08 (0.47–2.26) | Embolisation 24 (18.9) Surgical resection 7 (5.5) Radiosurgery 5 (3.9) Embolization + RS 6 (4.7) Embolization + Surgery 3 (2.4) Embolization + surgery + RS 1 (0.8) | 55 (43.3) | 40 (31.5) | 28 (22) | 2.74 (0.1–13) | 22 (16 to 30) | Min. 36 | 104 radiological FU | /90 who underwent angiography: 54 (60) | /104 with MRI follow-up: 72 (69.2) | 44 (43.9–53.1) | DSA: 90 MRI:104 | 11 (8.66 %) Annual bleeding rate 2.1 %. | — | 8 (6.3%) permanent FND and 6 (4.9 %) radiation necrosis | 15 (12.3) | 2 (1.6 ) | Total 5 (4.1). 5/5 ICH | — | — |
| Han *et al.* 2008 [31] | 218 | 73 (33.49) | Mean: 31 (±SD 15) | Peripheral 151 (69.3) Deep 50 (22.9) Cerebellum 17 (7.8) | — | I 54 (24.8) II 79 (36.2) III 68 (31.2) IV 15 (6.9) V 2 (0.9) | — | — | 20 (9.2) | — | Embolisation 32 (14.7%) Surgical resection, evacuation of ICH from AVM and Aneurysm occlusion 33 (15.14) | 106 (48.6) | 58 (26.6) | 15 (6.9) | 3.4 (0.17–35.2) | 18.0 (10.0–25.0) | Mean: 44 (±SD 20) | 218 | Min. 2 year follow-up /119: 79 (66.4) Angio rate; 79/218 total or 79/119 who underwent angiography at min. 2 years. | Min. 2 year follow-up: 131 / 183 (71.6) | 24 | Angiography: 119 MRI: 183 | 12 (5.5%) annual bleeding rate 1.9%. Four of these patients had permanent neurological deficits and 1 died. The neurological deficits in 7 of the patients improved completely within 2 years. | Total[g]: /157 with regular MRI FU: 30 (19.1) including symptomatic 9 (5.73) | 8 (5.1%) | — | 0 (0) | Total 4 (1.83). 1/4 ICH 3/4 Unrelated | 24 (11) | — |
| Hasegawa *et al.* 2018 [32] | 592 | 248 (42%) | 38 (20–80) | Deep[e] location 217 (37) | 338 (57) | I–II 328 (55%) | Deep: 308 (52) Superficial 284 (48.0) | 2.2 (0.5–6) | — | — | Embolisation 63 (11%) Surgical Resection 52 (9%) | 311 (53) | — | — | 2.7 (0.1–44.5) | 20 (15–28) | 92 (1–320) | 592 | 391 (66). Actuarial obliteration rates at 2/3/4/6 years were 23/53/73/85% | 391 (66). Actuarial obliteration rates at 2/3/4/6 years were 23/53/73/85% | 30 | Angiography: DSA | 38(6.4%). Actuarial haemorrhage rate at 1/2/5/10 years were 2.9/4.9/ 5.5/6.4%. | Total: 1/2/3 years rate of 15/30/32% | Permanent symptomatic at 2/5/10/15 years of 3/4/4/7% | — | 35(5.9) including encapsulated haematoma | — | 37 | — |
| Hirschmann *et al.* 2019 [35] | 265 | 124 (47%) | 40 (7–80) | — | 144 (54%) | Spetzler-Ponce A 130 (49%) B 109 (41%) C 26 (10%) | Deep 165 (62%) Superficial 100 (38%) | — | — | 1.3 (0.2–4.2) | Embolisation 19 (7.16%) Surgical Resection 8 (3.02%) Repeat GK 68 (26%) | 81 (31) | — | — | 2.3 (0.1–42.0) | 19 (11–22) | 66 (7.2–384) | 265 | — | Actuarial obliteration rate at 3/5/10 years were 51/63/74% | 45.6 | Angiography and MRI | 24 /265 (9%) Annual haemorrhage rate 1.3%. | — | Total permanent: 13/265 (4.9%) **includes the occurrence of radiologically diagnosed edema or late onset cyst formation with or without new neurological symptoms | — | see transient RIC column | Total: 7 (1.5) | 49 (18.9) | — |
| Hu *et al.* 2020 [36] | 98 | 38 (38.8) | 37 (4-80) | Lobar 81 (82.7) Deep location 29 (29.6) -Thalamus, basal ganglia 3/98 -Brainstem 7/98 Cerebellum 8 (8.2) | 61 (62.2) | I 17 (17.35) II 33 (33.7) III 33 (33.7) IV 14 (14.29) V 1 (1.02) | Deep 57 (58.2) Superficial 41 ((41.8) | 2.58 | 25 (25.5) | — | 0 (0) | 47 (48.0) | 29 (29.6) | — | 7.1 (0.1-42.6) | 18 (16-21) | 47.5 (3-93) | 98 | 43 /98 (43.9) | 63 (64.3) | 31 (14-82) | Angiography: 43 (43.9%) MRI only: 20 (20.4%) | 2 (2%) | Total: 62(63.3%) including symptomatic 7 (7.1%) | — | — | — | — | — | — |
| Izawa *et al.* 2005 [39] | 237 | 93 (39.2%) | mean: 30.1 (6–74) | Lobar 159 (67%) Deep 65 (27.4) Cerebellum 13 (5.5%) | — | I 33 (13.9%) II 69 (29.1%) III 104 (43.9%) IV 31 (13.1%) | — | — | — | — | — | 130 (54.9) | 50 (21.1) | — | mean: 4.7 (0.1–28.5) | mean: 20.2 (12–26) | 81.6 (24–150) | 237 | 130 (54.9) | 130 (54.9) | — | Angiography | 8 (3.4%) | — | Total: 1 (0.4%) including symptomatic 0 (0) | 4 (1.7) | 8 (3.4) | Total: 3 (1.27%). 3/3 ICH | — | — |
| Kano *et al.* 2014 [41] | 474 | 222 (46.8) | 33 (±SD 1.32) | Cortical 250/474 (52.7) Deep 169 (35.7) -Thalamus 64/474 (13.5) -Brain stem 56/474 (11.8) -Basal ganglia 49 (10.3) cerebellum 34 (7.2) Corpus callosum 14 (3.0) ventricles 3 (0.6) | 430 (90.7) | III 474 (100) | Deep 326 (68.8) Superficial 148 (31.2) | 1.9 (0.6–2.9) | 32 (6.8) | — | Embolisation alone: 67 (14.1%) Surgical resection alone: 44 (9.3%) Embolisation + resection: 14 (3) | 268 (56.5) | — | — | 3.8 (0.1–26.3) | 20 (13–25) | 89 (2–278) | | Actuarial obliteration: 3/4/5/10 were 39%, 57%, 59%, 62%. | Actuarial obliteration: 3/4/5/10 years were 48%, 69%, 72%, 77% | — | Angiography: 198 MRI only:258 | 38 (8%). Annual hemorrhage rate 2.7% (1385 patient-years) | symptomatic 17 (3.58) | 13 (2.74%) Sx | — | 9 (1.9) | Total: 21 (4.4). 20/21 ICH 1/21 ARE | 59 (12.4) | 34/59 (58) obliteration |
| Kano *et al.* 2014 [43] | 133 | 63 (47.4) | 26 (3–69) | Deep 133 (100) -Basal Ganglia 56 (40) -Thalamus: 77 (55) | — | II 11 (8.3) III 48 (36.1) IV 20 (15.0) VI 54 (40.6) | — | 2.0 (0.6–4.8) | 7 (5%) | 1.39 (±SD 0.56) | Embolisation 18 (13.5) Surgical Resection 20 (15.0). | 113 (85) | 3 (2) | 3 (2) | 2.7 (0.1–20.7) | 20 (15–25) | Clinical: 81(2–265) Radiological: 61 | | Actuarial obliteration 3/4/5/10 years were 47%/60/ 63/63. | Actuarial obliteration 3/4/5/10 years 57%/ 70/72/72 | DSA: 36.7 (95% CI 29.19–44.2) | Angiography: 63 MRI only:78 | 15 (11%). Annual hemorrhage rate 4.7% (364.4 patient-years) | symptomatic 13 (9.8%) | 6 (4.5%) Sx | — | 1 (0.8) | Total: 11 (8.3). 7/11 ICH 4/11 Un-related | 18 (13.5) | 9/18 obliteration |
| Kano *et al.* 2014 [44] | 67 | 25 (37.3) | 41 (6–79) | Deep 67 (100) Midbrain 40 (59.7) Pons 32 (47.8) Medulla 3 (4.5) | 67 (100) | III 7 (10.4) IV 0 (0) V 0 (0) VI 60 (89.6) | — | — | 7 (10.4) | 1.45 (±SD 0.55) | Embolisation: 11 (16.4) | 51 (76) | 0 (0) | 4 (6) | 1.4 (0.1–13.4) | 20 (14–25.6) | 73.3 months (6–269) | | Actuarial obliteration 3/4/5/10 years were 36%/ 63/63/63. | Actuarial obliteration 3/4/5/10 years were 41%/ 70/70/76 | DSA:41.3 (95% CI 35.73–46.87) | Angiography: 28 MRI only:35 | 4 (6%). Annual hemorrhage rate 1.9% (206.7 patient-years) | Symptomatic 4 (6.0) | 7 (10.4) Sx | — | 2 (3.0) | Total: 10 (14.9). 4/10 ICH 1/10 hydrocephalus 5/10 unrelated | 4/67 (6.0) | 1/4 obliteration |
| Kano *et al.* 2012 [45] | 217 | 106 (49) | 38 (3–77 | Cortical 174 (80.2) Deep 9 (4.1) -thalamus 7 (3.2) -basal ganglia 2 (0.9) pineal region 1 (0.5) intraventricular 4 (1.8) cerebellum 19 (8.8) corpus callosum 10 (4.6) | — | I 34 (16%) II 183 (84%) | — | 1.9 (0.5-3.77) | 21 (9.7) | 1.12 (±SD 0.52) | 0 (0) | 78 (36) | 61 (28) | 30 (14) | 2.3 (0.1–14.1) | 22 (15–27) | 64 (6–267) | | Actuarial obliteration rates: 3/4/5/10 years were 41%, 66%, 77%, and 83%. | Actuarial obliteration: 3/4/5/10 years were 58%/ 87/90/93 | DSA: 37 (95% CI 36–39). Obliteration time defined as halfway time-point between last MRI image and documented MRI/DSA obliteration | Angiography: 100 MRI only:148 | 13 (6%) Annual hemorrhage rate 2.3% (559.1 patient-years) | Total: 5 (2.3%) including symptomatic 5 (2.3%) | 0 Permanent deficit 0 Radiation necrosis | 3 (1.4) /5 who experienced transient symptomatic RICs | 2 (0.9) | Total: 6 (2.8). 6/6 ICH | 8 (3.7) | 4/8 obliteration |
| Kim BS *et al.* 2019 [46] | 264 | 98 (37.1) | Mean: 40.7 (±SD 12.0) | Supratentorial lobar 238 (90.2) Deep 7 (2.6) -Thalamus 4 (1.5) -Basal ganglia 3 (1.1) Corpus callosum 3 (1.1) Cerebellum 8 (3.0) Insula 5 (1.9) Intraventricuar 3 (1.1) | 121 (45.8) | I 52 (19.7) II 87 (32.9) III 87 (32.9) IV 38 (14.4) | Deep 65 (24.6) Superficial 199 (75.1) | 3.6 (±SD 1.6) | 53 (20.1) | Mean: 1.35 (0.46–3.54) | 0 (0) | 0 (0) | 66 (25.0) | 92 (34.8) | Mean: 4.8 (±SD 5.3) | Mean: 21 (12–30) | Mean: Clinical and Radiological were 55.5 and 44.7 (3.4–179.9) | 264 | 115 /264 (43.5) | 164 (62.1). Actuarial obliteration rates at 3/5/7/10 years: 37.1%/ 70.5/84.1/90.1 | 40.1 (95% CI: 37.0–45.5) | MRI alone: 49/164 (18.6%) DSA: 115/164 (43.5%) | 19 (8.26%) Annual Rate 2.85% (644.6 person-years) | Total: 149 (56.4%) including symptomatic in 35 (13.3%) | symptomatic 3 (1.1%) | 24 (9.1) | — | Total: 2 (0.76). 1/2 AVM-related causes, 1/2 unrelated | 34 (12.9) | — |
| Kiran *et al.* 2009 [47] | 53 | 12 (22.6) | Mean: 22.7 years (3–55) | Deep 53 (100) -Basal ganglia/ thalamus, 47; Brainstem, 6). | 53 (100) | I 0 (0) II 0 (0) III 40 (75) IV 11 (21) V 2 (4) | — | — | — | — | Embolisation 5 (9%) | 43 (81) | 8 (15) | — | Mean: 4.3 (0.1–36.6) | Mean: 23.3 (16–25) | Mean: 28 (12–96) | 53 | Min. 4 year angiography follow-up /19: 14 (74%) Actuarial obliteration rates, 3 and 4 years: 68 and 74% | — | — | Angiography: 19 | 5 (9.4%) | Symptomatic 4 (7.54) | symptomatic 4 (7.54) | — | — | — | — | — |
| Liscák *et al.* 2007 [49] | 330 | 152 (46.5%) | 35 (3-78) | Lobar 250 (75.76%) Deep 42 (12.73%) Cerebellum and Corpus callosum 38 (11.52%) | — | I 39 (11.8%) II 156 (47.3%) III 100 (30.3%) IV 35 (10.6%) V 0 (0) | — | 2.2 (0.6-6.1) | — | — | Embolisation 58 (17.6%) Surgical resection 64 (19.4%) fractionated radiotherapy 6 (1.8%) Resection+Embolisation 7 (2.12%) | 207 (63) | 105 (31.82) | — | 3.9 (0.15-28.6) | 20 (8-32) | 38 (1-118) | 300 | 222/300 (74) | 222/300 (74) | 25 (12-96) | Angiography 222 | 16/300 (5.33%) | — | 8/300 (2.67%) | 12/300 (4) | — | — | 68/300 (22.67) | 47/68 (69) |

| Study, year of publication | Total number of patients | Female patients (%) | Median age (range/ SD) | Location[2] (%) | Eloquent Location (%) | SM grade (%) | Venous Drainage (%) | Median Maximum Nidus Diameter, cm (range) | Associated flow-related or intranidal aneurysm (%) | Median RBAS OR VRAS Score | Pre-GKRS intervention (%) | Presentation with Haemorrhage (%) | Presentation with seizure (%) | Asymptomatic presentation (%) | Median Nidus Volume cm$^3$ (range) | Median Prescription dose Gy (Range) | Median Duration of follow-up months(range) | Number of patients with follow-up | Complete Obliteration rate — Angiography-confirmed (%) | Complete Obliteration rate — Angiography or MRI confirmed (%) | Median Time to obliteration (months) | Method of determining obliteration ± no. of patients underwent imaging modality | Post-GKRS Haemorrhage (%) | Post-GKRS transient RIC (radiological or symptomatic) | Post-GKRS permanent RIC (radiological or permanent FND) | Post-GKRS new onset/ increase frequency seizures | Cyst formation (%) | Post-GKRS Mortality (%) | Patients who underwent repeat-GKRS (%) | Outcomes for repeat-GK cohort |
|---|---|---|---|---|---|---|---|---|---|---|---|---|---|---|---|---|---|---|---|---|---|---|---|---|---|---|---|---|---|---|
| Matsunaga et Shuto. 2014 [51] | 82 | 28 (34.1) | mean 43.3 (±SD17.5) | Deep 27 (32.9) cerebellar vermis 20 (24.4) cerebellar hemispheres 35 (42.7) | 60 (73.2) | I 14 (17.1%) II 37 (45.1%) III 30 (36.6) IV 1 (1.2%) | — | 1.7 (0.4-5.5) | 12 (14.6) | 1.42 (0.58-3.22) | embolisation 11 (13.4%) surgical resection 5 (6.1%) | 69 (84.1) | 0 (0) | 11 (13.4) | 0.95 (0.03-22.9) | 18 (12-25) | 73.2 (60-219.6) | 82 | 41 (50) | 65 (79.3) Actuarial rate at 3/5 years is 58.5% and 78% | 26.4 (6-63.6) | Angiography: 41 MRI: 24 | 2 (2.43%) Annual bleeding risk post GK was 1.2% at 1 year and 0.49% at 5 years. | Total: 20 (24.4%) patients including symptomatic in 6 (7.32%) | symptomatic 6 (7.31%) 1 radiation necrosis (1.2) | — | 0 (0) | — | 5 (6.1) | 2/5 obliteration at 3 and 3.2 years post repeat-GK |
| Missios *et al.* 2014 [52] | 165 | 82 (53.9) | Mean: 43.6 (±SD 17.4) | Cortical 125 (75.75) Deep 23 (13.9) -brainstem 9 (5.5) -thalamus/ basal ganglia 14 (8.5) cerebellum 13 (7.9) Other 4 (2.4) | — | I 11 (6.7) II 67 (40.6) III 69 (41.8) IV 18 (10.9) | — | Mean: 2.74 (0.8–5.6) | 22 (13.3) | 1–2 104 (63.1) | Embolisation 0 (0) Surgical resection 5 (3.0) | 41 (24.8) | 36 (21.8) | 22 (13.3) | Mean: 6.3 (0.05–46.8) | Mean: 17.9 (10–28) | Mean: 36.2 (1–158) | 165 | 70 (42.42) Actuarial rates for 3, 4, 5 years are: 44%/ 54/66 | — | 40 months (95% CI 28.4–51.6) | Angiography | 7 (4.2%) | symptomatic 14 (9.03%) | — | 8 (4.8) | — | Total: 3 (1.81). 1/3 ICH 1/3 cerebral edema 1/3 intractable seizure | 38 (23.0) | — |
| Nicolato et al. 2002 [54] | 242 | 102 (42.1) | Mean: 33.2 (5-74) | Cortical 209 (86.4) Deep 33 (13.6) -Basal Ganglia 33 (13.6) | — | I 14 (5.7) II 134 (55.4) III 89 (36.8) IV 5 (2.1) | — | — | — | — | Embolisation 116 (47.9) Surgical resection 9 (4.5) Previous RT 2 (1) | 137 (56.6) | 75 (36) | 16 (8) | 3.12 (0.1-10) | 22.9 (14-28) | Not stated-"minimum 6 months" | 191 | 111/131 (84.8) 131 had minimum 2 years follow-up. | 111/131 (84.8) 131 had minimum 2 years follow-up. | Mean: 27.7 (6.2-76.5) | Angiography 131 | 5/191 (2.0.7%) | 8/191 (4.2) | 4/191 (2.1) | — | — | total 2/191 (1.04). 1/2 ICH 1/2 unrelated. | — | — |
| Orio *et al.* 2006 [56] | 100 | 48 (52.7) | 33 (9–66) | Cerebrum 91 (91%) Deep 9 (9.9) -Basal Ganglia 4 (4%) -Thalamus 3 (3%) -Brainstem 2 (2%) | — | *d* | — | — | — | — | Previous LINAC: 13 (14.3%) Repeat GK 4 (15.8%) | 40 (44) | — | — | 4.3 (0.2–27.2) | 20 (13–24) | 32 | 91 | Actuarial obliteration rates: 3/5 year of 21/ 72% | Actuarial obliteration rates: 3/5 year of 21/ 72% | — | Angiography and MRI | 12(12%) | — | 8 (8%) including 5 radiation necrosis (5.5) and 3 symptomatic (3.3) | — | — | Total: 3 (3.3). 3/3 ICH | 18 (20) | Overall primary and secondary GK: 3/5 year obliteration rates of 21 and 72% |
| Pan *et al.* 2000 [57] | 240 | 104 (43.3) | Mean: 28 (2–77 years) | Lobar 133 (55) Deep 58 (24) -Basal ganglia 24 (10) -Brainstem 34 (14) corpus callosum 20 (8.3) cerebellum 21 (8.8) lat ventricle 8 (3.3) | — | I 8 (3.3) II 48 (20) III 87 (36.3) IV 74 (30.8) V 20 (8.3) VI 3 (1.25) | — | 6.15 | — | — | Embolisation: 5 (2.1) Surgical resection 33 (13.8) Radiotherapy 3 (1.25) | 178 (74) | 70 (29) | — | 6.15 (±SD 8.01) | Mean: 19.1 | 26 (12–73) | 240 | /131 who underwent angiography: 115 (88). Actuarial obliteration rate 40 months is 75% | /131 who underwent angiography: 115 (88). Actuarial obliteration rate 40 months is 75% | — | Angiography: 131 | 10 (4.1%) | Total: 115 (47.9) including symptomatic 7 (2.9) | Symptomatic 8 (3.3) | — | — | Total: 1 (0.42) | 15 (6.3) | Obliteration 9/15 |
| Parkhutik *et al.* 2013 [58] | 102 | 49 (48%) | 34 years (IQR 22–50) | — | — | I 35 (34 %) II 34 (33%) III 26 (25 %) IV 8 (8%) | Deep 42 (41.2) Superficial 60 (59) | 1.5 | 14 (14) | — | Embolisation 66 (64.7 %) Surgical resection 2 (2.0%) Endovascular and Surgical resection 1 (0.98%) | 41 (40) | 36 (35) | — | 3.95 (IQR 0.95–5.9) | Mean: 18.5 (12–24) | 63 months (IQR 35–103) | 102 | /82 who had 3 year angiographic follow-up: 50 (79) | /82 who had 3 year angiographic follow-up: 50 (79) | — | Angiography: 63 patients | 3 (2.9%) | Total: 17[b] (16.6 %) | Necrosis 7 (6.9 %) | 2 (1.9) | *Cyst incidence included in necrosis* | Total: 4 (3.9). 2/4 ICH and 2/4 of unrelated causes. | 19 (18.6) | 11/19 |
| Pollock *et al.* 2016 [64] *1990-1999 cohort* | 160 | 84 (52.5) | 36 (3-82) | Deep 32 (20.0) | 128 (80.0) | Spetzler-Ponce A 52 (32.5) B 73 (45.6) C 35 (21.9) | — | 2.8 (0.9-6.2) | — | 1.46 (0.41-5.92) | Embolisation: 12 (7.5) Surgical Resection: 15 (9.4) | 55 (34.4) | — | — | 5.7 (0.4-45.8) | 18.0 (15.0-22.0) | Clinical: 93 (3-290) | 160 | | 130/160 (81.3). 4-year and 8-year obliteration rate of group 1 patients was 67.9% and 86.1%, | — | Angiography and MRI | 37 (9.7%). Annual hemorrhage rate 1.3% (2966 person-years) | — | Symptomatic 23 (14.4%) | 4 (1.05) /23 who experienced permanent RIC | — | Total: 14 (3.7). 13/14 ICH 1/14 radiation necrosis. | 26 (16.3) | — |
| Pollock *et al.* 2016 [64] *1999-2009 cohort* | 221 | 128 (57.9) | 42 (5-71) | Deep 30 (13.6) | 165 (74.7) | Spetzler-Ponce A 107 (48.4) B 85 (38.5) C 29 (13.1) | — | 2.4 (0.6-6.1) | — | 1.31 (0.21-4.40) | Embolisation: 8 (3.6) Surgical resection: 15 (6.9) | 63 (28.5) | — | — | 3.6 (0.1-35.4) | 20.0 (15.0-25.0) | Clinical: 93 (3-290) | 221 | | 168/221 (76.0). 4-year and 8-year obliteration rate 52.7% and 84.3% | — | Angiography and MRI | 37 (9.7%). Annual hemorrhage rate 1.3% (2966 person-years) | — | Symptomatic 8 (3.6%) | 4 (1.05) /23 who experienced permanent RIC | — | Total: 14 (3.7). 13/14 ICH 1/14 radiation necrosis. | 38 (17.2) | — |
| Raboud *et al.* 2018 [65b] | 64 | 32 (50.0) | Mean: 46 (13–79) | — | — | I 9 (14.3) II 14 (22.2) III 29 (46.0) IV 7 (11) V 4 (6.3) | — | — | — | 1.01–1.5 29 (46.0) | Embolisation 16 (25.0) Surgical resection 1 (1.6) Previous Gk 2 (3.1) LINAC 7 (10.9) | 32 (50.0) | 12 (18.8) | 6 (9.4) | 1.2 (0.032–11.3) | 24 (18–24) | 38 (12–75) | 64 | 30 /64 total (46.9) 30 /55 who underwent DSA (54.5) | 39 (61) Actuarial rates at 3/4/5years were 37.4/63.6/78.4%, | Mean: 35 (8–56) | Angiography: 55 MRI only: 9 | 3 (4.7%) | Total: 1 (1.56%) including symptomatic in 1 (1.56%) | 0 | 0 | 1 (1.6) | Total: 1 (1.75). 1/1 ICH | — | — |
| Tuleasca *et al.* 2020 [71] | 149 | 82 (55) | 40 (18–68) | Supratentorial lobar 128 (85.9) Deep 7 (4.7) -Thalamus 6 (4) -Brainstem 1 (0.7) Infratentorial 8 (5.4) Ventricular 6 (4) | 95 (63.8) | I 42 (28.2%) II 71 (47.7) III 36 (24.2) IV 0 (0) | Deep 32 (21.5) Superficial 117 (78.5) | — | 10 (6.7) | 2002 Median: 1.52 (0.4–2.9) | Embolisation 0 (0) | 0 (0) | 66 (44.3) | 32 (21.5) | 2 (0.09–10) | 24 (18–25) | 48 (12–154) | 149 | — | 104 (69.8) Actuarial obliteration at 3/4/5/6/8 years were 37.9%/ 55.6/ 63.3/72/76 | 36 (12–96) | MRI only: 30 (20%) Angiography: 119 (80%) | 3 (2%) | Total: 20 (13.4%) | symptomatic 5 (3.4%) | 1 (0.7%) | — | — | — | — |
| Zeiler *et al.* 2011 [74] | 41 | 17 (41.5%) | 40.9 (14-74) | Cortical: 34 (82.9) Deep 8 (19.5) -Brainstem 2/41 (4.9) -Basal ganglia 6/41 (14.6) Cerebellar 4 (9.8) | — | I 7 (17.1%) II 9 (22.0%) III 21 (51.2%) IV 4 (9.8%) V 0 (0) | — | 2.36 | — | 1.68 | Embolisation 1 (2.44%) Embolisation and partial resection 1 (2.44%) Repeat GK 7 (17.1) | 18 (43.9) | 24 (58.5) | 9 (22.0) | 5.05 cm3 | 20.3 (16 – 26.4 ) | 43.1 | 41 | | MRI OR CTA OR DSA: 36 (87.8) | 27.6 | MRI OR CTA AND DSA: 20 MRI OR CTA: 16 | 2 (4.88%) | Total: 11 (26.8%) including symptomatic in 7 (17.1%) | 1 (2.44%) | — | — | 3 (7.32) | 5 | 1/5 obliteration |
| Zhao *et al.* 2008 [75] | 341 | 106 (31.1) | Mean: 24.2 (1.1 - 79) | Lobar 243 (71.3%) Deep 47 (13.8) -Basal ganglia 19 (5.6%), -Thalamus 14 (4.1%), -Brain stem 14 (4.1%). Corpus callosum and ventricular 26 (7.6%) Cerebellum 25 (7.3%) | — | *d* | — | — | — | — | Embolisation 54 (15.8%) Surgical Resection 26 (7.6%) | 171 (50.2) | 78 (22.9) | 24 (7.0) | Mean: 5.4 | Mean: 20.5 (10 to 30) | 76.8 (±SD 16.8) | 341 | — | 267 (78.4) (follow-up 18 months +) | — | DSA (31/341) MR/MRA (298/341) CT (12/341) | 10 (2.9%) | Symptomatic 12 (3.52%) | symptomatic 3 (0.88%) | — | 2 (0.6) | Total: 4 (1/17). 3 ICH 1 ARE | 3 (0.9) | — |

Abbreviations: AVM = arteriovenous malformation; EBRT = fractionated external beam radiation therapy; Magnetic Resonance Imaging (MRI); RBAS = modified Radiosurgery Based Arteriovenous malformation Score; SM = Spetzler-Martin; Stereotactic Radiosurgery (SRS); Virginia Radiosurgery AVM Score (VRAS)— or NA = Data not available.

[1] explicitly not stated that there were no mortalities secondary to GKS treatment;[2]Eloquent locations, as defined by the Spetzler-Martin (SM) grading system, included sensorimotor, language, and visual cortex, hypothalamus and thalamus, internal capsule, brain stem, cerebellar peduncles, and deep cerebellar nuclei. Deep locations, as defined by the modified radiosurgery-based AVM score (RBAS), included the thalamus, basal ganglia, and brain stem, unless stated otherwise. [a] Chang *et al.* 2000 [6]: Brainstem-located AVMs have been totalled alongside author's definition of deep location which includes the thalamus, lentiform nucleus, caudate nucleus, and internal capsule but excludes brainstem [b]Defined "major radiological injury" that included moderate or severe edema and radiation necrosis (excluded minor/perilesional oedema). Was not possible to reliably extract whether RICs were transient or permanent so excluded from final statistical analysis. [d]Spetzler martin grade could not be calculated (venous drainage not stated). [e]Hasegawa *et al.* 2018 [32]: Deep location includes basal ganglia, thalamus, brainstem, cerebellum, interventricular regions, and corpus callosum. [f]Han *et al.* 2008 [31]. Deep location includes corpus callosum (in addition to Basal Ganglia/Brainstem/Thalamus). [g]Han *et al.* 2008 [31]. classified 'severe' post-radiosurgery injury as significant and this has been reflected in the data included in this analysis. [h]Superficial includes cerebral hemispheres, lateral ventricles, parasagittal/paraventricular regions, corpus callosum, cerebellar tentorium.

**APPENDIX 7. Summary of methodological characteristics of the included studies**

| | |
|---|---|
| *Study set-up* | |
| Multi-center | 1 (3%) |
| Single-center | 33 (97%) |
| *Continent (Single-centre only)* | |
| North-America | 13 (39%) |
| Europe | 8 (24%) |
| Asia | 12 (36%) |
| *Reporting of the selection criteria of the study* | |
| Specified | 23 (68%) |
| No specified | 33 (32%) |
| *Participant identification* | |
| Retrospective | 33 (97%) |
| Prospective | 1 (3%) |
| Unspecified | 0 (0%) |
| *Reporting of consecutive patients* | |
| Yes | 8 (24%) |
| No | 7 (21%) |
| Not specified | 19 (56%) |
| *Participant follow-up* | |
| Retrospective | 33 (88%) |
| Prospective | 4 (12%) |
| Unspecified | 0 (0%) |

**APPENDIX 8. Moderator analysis/Meta-regression**

| Characteristic | Haemorrhage | PRIC | TRIC | Angio-obliteration | Angio/MRI-obliteration |
|---|---|---|---|---|---|
| Age, % | | | | 0.018 | |
| Male, % | | | | | |
| Study Midyear | | | | 0.021 | |
| Margin Dose, Gy | | | | | |
| AVM Volume, cm³ | | | | | |
| Deep venous drainage, % | 0.005 | | | | 0.035 |
| Eloquent location, % | 0.026 | | | | |
| Deep location, % | | 0.005 | 0.002 | | |

Cells shaded green indicate statistical significance ($P<0.05$) with actual values of significance stated within corresponding cells.

# APPENDIX 9. Funnel Plots

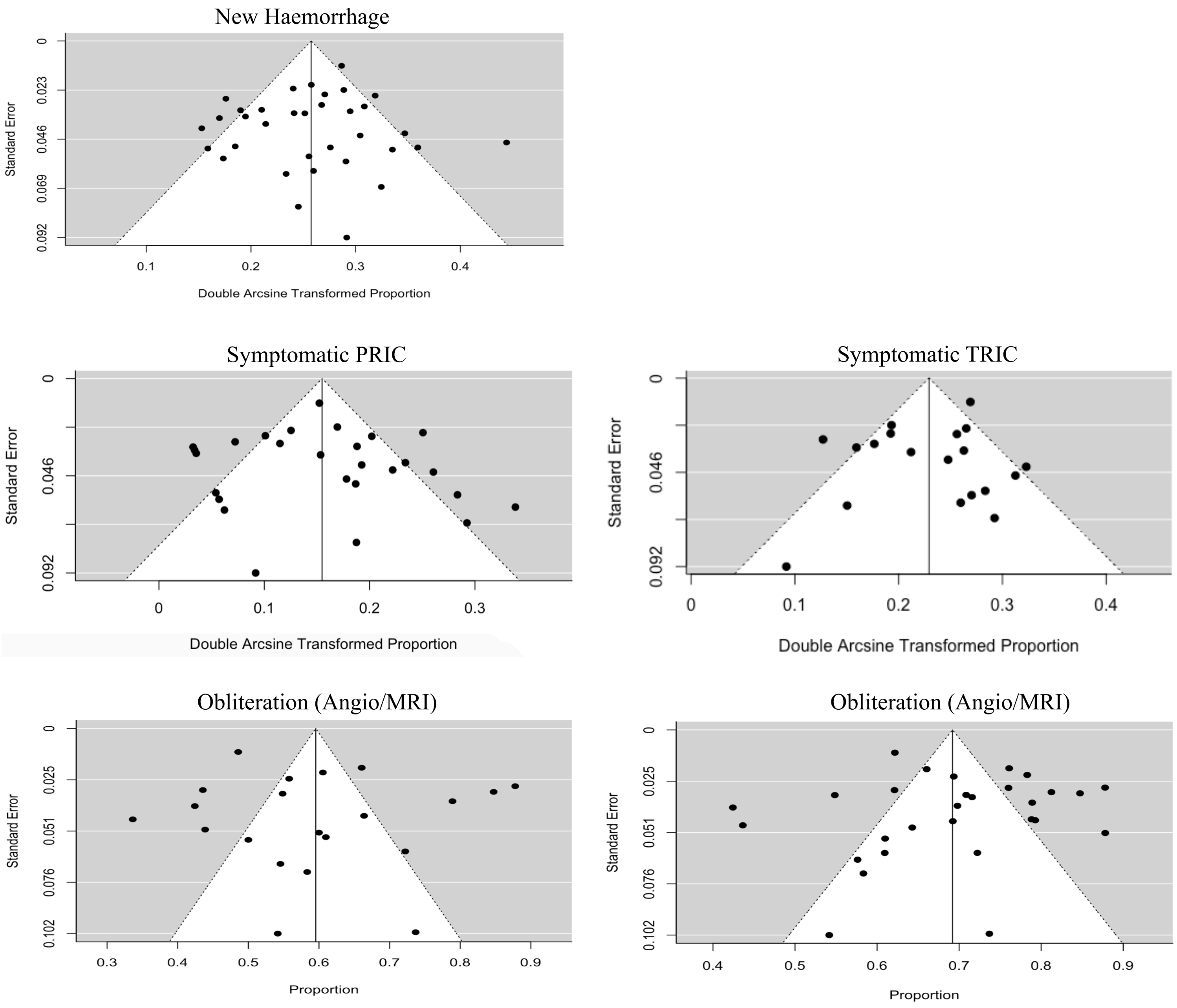

Supplement: Supplementary file 1 — Supplementary file1 (PDF 764 kb) [file 10143_2022_1751_MOESM1_ESM.pdf]
